# Characterization of the early molecular changes in the glomeruli of *Cd151*^−/−^ mice highlights induction of mindin and MMP-10

**DOI:** 10.1038/s41598-017-15993-3

**Published:** 2017-11-22

**Authors:** Crystal Naudin, Brian Smith, Danielle R. Bond, Matthew D. Dun, Rodney J. Scott, Leonie K. Ashman, Judith Weidenhofer, Séverine Roselli

**Affiliations:** 1 School of Biomedical Sciences and Pharmacy, Faculty of Health and Medicine, University of Newcastle, Newcastle, New South Wales Australia; 2 Hunter Medical Research Institute, New Lambton, New South Wales Australia; 3 Emory University, Atlanta, Georgia USA; 4 School of Mathematics and Physical Sciences, University of Newcastle, Newcastle, New South Wales Australia; 5 Hunter Area Pathology Service, John Hunter Hospital, New Lambton, New South Wales Australia

## Abstract

In humans and FVB/N mice, loss of functional tetraspanin CD151 is associated with glomerular disease characterised by early onset proteinuria and ultrastructural thickening and splitting of the glomerular basement membrane (GBM). To gain insight into the molecular mechanisms associated with disease development, we characterised the glomerular gene expression profile at an early stage of disease progression in FVB/N *Cd151* ^−/−^ mice compared to *Cd151* ^+/+^ controls. This study identified 72 up-regulated and 183 down-regulated genes in FVB/N *Cd151* ^−/−^ compared to *Cd151* ^+/+^ glomeruli (p < 0.05). Further analysis highlighted induction of the matrix metalloprotease MMP-10 and the extracellular matrix protein mindin (encoded by *Spon2*) in the diseased FVB/N *Cd151* ^−/−^ GBM that did not occur in the C57BL/6 diseased-resistant strain. Interestingly, mindin was also detected in urinary samples of FVB/N *Cd151* ^−/−^ mice, underlining its potential value as a biomarker for glomerular diseases associated with GBM alterations. Gene set enrichment and pathway analysis of the microarray dataset showed enrichment in axon guidance and actin cytoskeleton signalling pathways as well as activation of inflammatory pathways. Given the known function of mindin, its early expression in the diseased GBM could represent a trigger of both further podocyte cytoskeletal changes and inflammation, thereby playing a key role in the mechanisms of disease progression.

## Introduction

Glomerular diseases are characterised by proteinuria due to damage of the glomerular filtration barrier, initiated either by an intrinsic genetic defect or as a consequence of an underlying systemic disease. Acting as the selective filter of the kidney, the glomerular filtration barrier consists of a fenestrated endothelium, a highly specialised epithelium consisting of podocytes, and an intervening glomerular basement membrane (GBM). Podocytes are composed of a cell body extending into primary branches that attach onto the GBM through secondary extensions called foot processes tightly interlinked by modified adherens junctions called slit diaphragms connecting to and regulating the actin cytoskeleton. Alterations in any component of the barrier, disrupting its tight organisation, lead to leakiness of the filter causing glomerular disease characterised by proteinuria that will ultimately progress with glomerulosclerosis that can result in end stage renal failure. The GBM is the initial site of injury in a large number of primary and secondary glomerular pathologies such as Alport syndrome and diabetic nephropathy, respectively^1,2^. In many glomerulopathies, however, the glomerular filtration barrier compartment in which the initiating damaging event occurs remains unknown. This is due to the fact that a functional defect in any of the three compartments of the filter will often converge onto similar pathways resulting in the same disease characteristics and histopathological features. In this regard, comprehensive analysis of single gene disorders and mouse models, such as *Cd151* knockout mice, is crucial to a better mechanistic understanding of the tightly orchestrated regulation of glomerular filtration in both normal circumstances and in the context of glomerular disease.

Rare genetic mutations in the *CD151* gene lead to nephrotic syndrome in humans, associated with thickening and splitting of the GBM, effacement of podocyte foot processes, interstitial nephritis and ultimately end stage renal disease^3^. Mice with a homozygous deletion of *Cd151* (*Cd151* ^*−/−*^) on the FVB/N genetic background display a similar kidney phenotype to humans with *CD151* mutations^4,5^. Our previous work has shown that disruption of GBM homeostasis and maturation as well as effacement of podocyte foot processes in FVB/N *Cd151* ^−/−^ mice are events that occur early in the course of disease^4^. Interestingly, this phenotype is highly dependent on genetic background, wherein on the original C57BL/6 background strain, *Cd151* ^−/−^ mice show a relatively mild phenotype displaying a hyperproliferative CD4^+^ and CD8^+^ T-cell response and defective keratinocyte migration, but normal kidney function^6^. CD151 is a transmembrane protein of the tetraspanin family^7^ that is broadly expressed by many cell types. Tetraspanins form dynamic membrane complexes with each other, bringing together tetraspanin-associated proteins such as integrins and kinases, enabling specific functions such as cellular signal transduction and cell-matrix adhesion^8^. Characteristically, CD151 is present on the basolateral surface of epithelial cells juxtaposed with basement membranes, where it has strong associations with the α6β4 and α3β1 laminin-binding integrins^9–16^. We have previously shown that *CD151* is expressed at the base of podocyte foot processes, along the GBM, where it colocalises with α3β1^4,17^. Furthermore, via its interaction with α3β1 integrin, CD151 strengthens the anchorage of podocyte foot processes to the GBM, especially under high pressure conditions^18^.

Interestingly, the GBM abnormalities observed in humans and FVB/N mice lacking CD151 are reminiscent of Alport syndrome, a genetic disorder resulting in an abnormal type IV collagen network and a GBM with laminar or “basketweave” appearance, previously considered unique to this disease^1^. This suggested that FVB/N *Cd151* ^−/−^ mice may represent a valuable model for the understanding of various forms of glomerular disease associated with GBM damage, regardless of the primary cause. In order to better understand the mechanisms involved in the tight regulation of the structure and function of the glomerular filtration barrier and potentially identify novel intervention points to prevent or slow glomerular disease progression, we further characterised the early gene expression changes associated with glomerular defect in 3-week-old FVB/N *Cd151* ^−/−^ mice. At this age, GBM maturation in wild type mice is complete and GBM and podocyte ultrastructural defects are evident in FVB/N *Cd151* ^−/−^ kidneys, whilst secondary changes such as glomerulosclerosis and inflammation are not yet prominent^4^. Using this model, we identified a large number of differentially expressed genes in FVB/N *Cd151* ^−/−^ glomeruli amongst which we highlighted the induction of MMP-10 and the extracellular matrix protein mindin in the Alport-like diseased FVB/N *Cd151* ^−/−^ GBM. Furthermore, enrichment of differentially expressed genes was shown to occur within the axon guidance, actin cytoskeleton and inflammation signaling pathways. Given the known cellular function of mindin, we propose that early mindin expression might be involved in triggering both further podocyte cytoskeletal changes and an inflammatory response, therefore playing a key role in the early mechanisms of glomerular disease progression.

## Results

### Genome wide gene expression analysis reveals significant changes in FVB/N *Cd151*^−/−^ compared to *Cd151*^+/+^ glomeruli

To further delineate the molecular mechanisms associated with damage of the glomerular filtration barrier upon *Cd151* deletion, we compared the whole genome expression profiles of FVB/N *Cd151* ^−/−^ and *Cd151* ^+/+^ glomeruli. We have shown previously that FVB/N *Cd151* ^−/−^ mice present with proteinuria associated with GBM damage shortly after birth, which becomes more severe at 3 weeks of age and is associated with some degree of effacement of podocyte foot processes, whereas glomerulosclerosis develops later in the course of the disease. In order to identify the early molecular events associated with glomerular disease in *Cd151* ^−/−^ mice thus avoiding the bias of secondary changes such as glomerulosclerosis, we performed gene expression experiments at this 3–4-week-old time point. As anticipated *Cd151* ^−/−^ mice showed complete abolition of the *Cd151* gene transcript representing the most significant change in the data set. Of the 18098 genes assayed, 8875 were detectable above background in at least one group of mice. Further *p*-value filtering of the normalised data and filtering for signal intensity ([n > 0] ≥50), to remove genes that were not detected at a minimum level in at least 1 sample, revealed 72 genes significantly up-regulated and 183 genes down-regulated in FVB/N *Cd151* ^−/−^ compared to *Cd151* ^+/+^ glomeruli (Supplementary Tables S2 and S3 respectively, p < 0.05).

**Figure 1:**
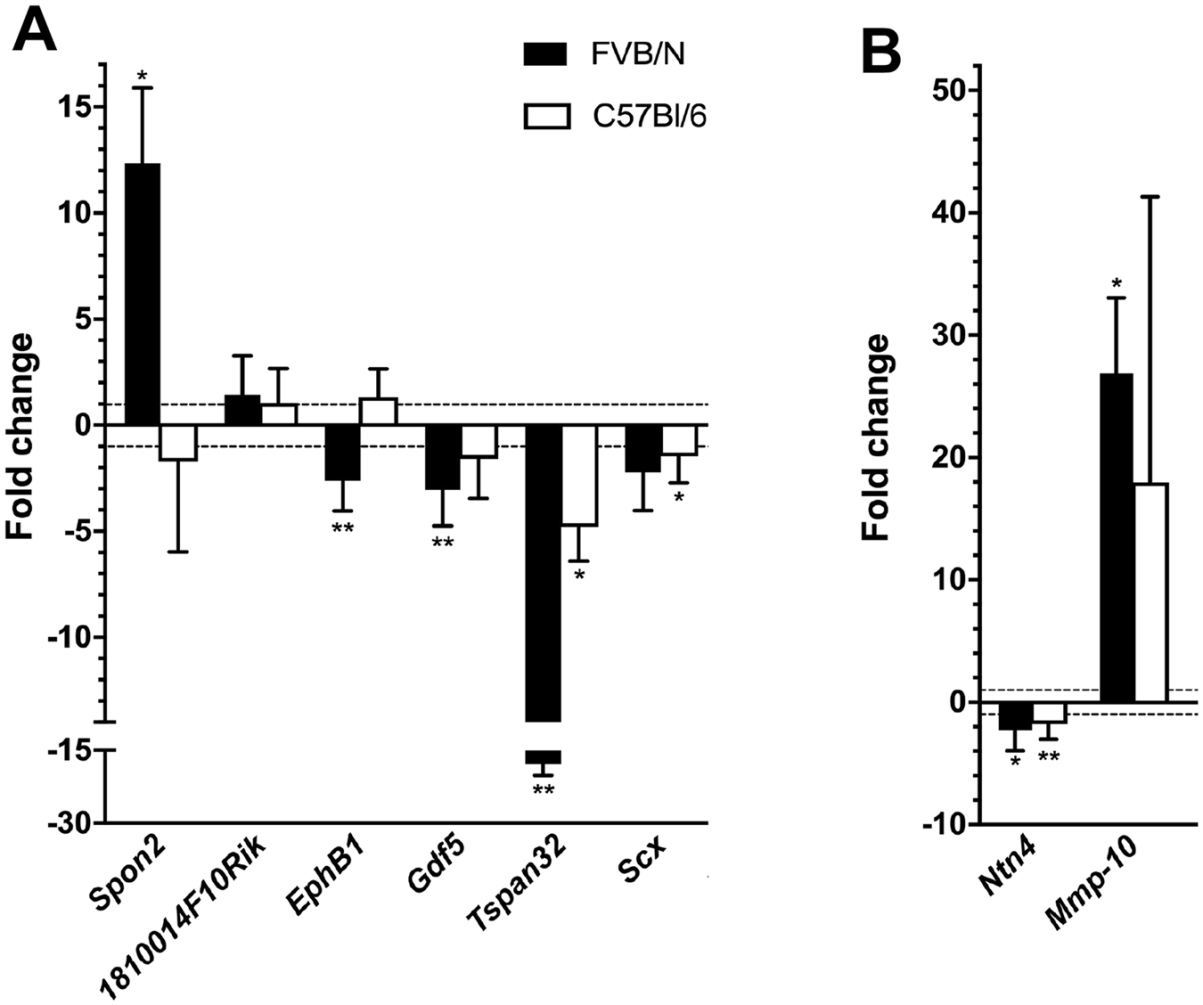
Real-time PCR validation of gene expression changes in *Cd151* ^−/−^ glomeruli relative to wild type controls. Real-time PCR validation was performed for selected differentially expressed genes identified from the microarray data. (**A**) The *Spon2*, *1810014F10Rik*, *Ephb1*, *Gdf5*, *Tspan32*, and *Scx* genes were selected from the SAM result for overall validation of the microarray. (**B**) After data mining of the microarray data, *Ntn4* was identified as a downregulated basement membrane gene and *Mmp-10* was identified as a potentially upregulated MMP gene. Both genes were further investigated with real-time PCR. Relative levels of transcripts are shown for FVB/N *Cd151* ^−/−^ versus *Cd151* ^+/+^ glomeruli as well as for C57BL/6 *Cd151* ^−/−^ versus *Cd151* ^+/+^ glomeruli. Data are presented as fold change in *Cd151* ^−/−^ glomeruli relative to *Cd151* ^+/+^ glomeruli (n = 4 per group, age 3 weeks), error bars represent standard deviation of the mean following bootstrapping. *P*-values were calculated using the student’s t-test comparing expression levels between mouse groups. *p < 0.05, **p < 0.01.

As an unbiased approach towards identification of the most statistically significant gene expression changes, we also used SAM software (Significance Analysis of Microarrays, v 3.0)^19^. This statistical approach in addition to identifying statistically significant results also takes into account a false discovery rate. The SAM analysis of the 8875 genes detectable on the microarray revealed 24 genes with significantly different expression in FVB/N *Cd151* ^−/−^ compared to *Cd151* ^+/+^ mice (Table 1, false discovery rate (FDR) <0.2). Surprisingly, out of the 24 genes, only one, *Spon2*, was up-regulated (5.5-fold) in *Cd151* ^−/−^ glomeruli, whereas the other 23 genes were down-regulated. Interestingly, *Spon2* encodes an extracellular matrix protein called mindin (also known as spondin 2) that is an integrin ligand^20^. The *Spon2* gene as well as five other genes (*1810014F10Rik, Ephb1*, *Tspan32*, *Scx*, and *Gdf5*) from the SAM list were included in a real-time PCR validation experiment of a second cohort of mice, based on their fold change and/or functional relevance. Briefly, *1810014F10Rik* encodes the fucose mutarotase enzyme, *Ephb1* encodes the tyrosine kinase receptor EphB1, and the *Tspan32* gene encodes a tetraspanin protein (tetraspanin 32 also known as Tssc6). Finally, both *Scx* (encoding scleraxis) and *Gdf5* (encoding growth and developmental factor 5) encode transcription factors that have been associated with the regulation of expression of basement membrane components^21,22^. The real-time PCR results were consistent with the microarray data in FVB/N *Cd151* ^−/−^ glomeruli for the majority of genes tested (4 out of the 6 genes, Fig. 1A). The changes in gene expression for *Spon2, Ephb1, Gdf5*, and *Tspan32* were confirmed by real-time PCR. The differential expression of *1810014F10Rik* and *Scx* was not confirmed by real-time PCR, although the decrease in *Scx* expression did approach significance (*p* = 0.06).

**Table 1:** Significant gene expression differences between FVB/N *Cd151* ^−/−^ and *Cd151* ^+/+^ glomeruli.

Gene name (*Symbol*)	FDR	Fold change (*p*-val)
Tetraspanin Cd151 (*Cd151*)	0	Abolished (0.000)
Tetraspanin 32 (*Tssc6*)	0	Abolished (0.001)
Spondin 2 or mindin (*Spon2*)	0	5.484 (0.002)
TNF receptor superfamily 22 (*Tnfrsf22*)	0	−3.361 (0.003)
Growth & developmental factor 5 (*Gdf5*)	0	−5.331 (0.000)
Fucose mutarotase, FucU (*1810014F10Rik*)	0	−19.367 (0.001)
Dihydrouridine synthase 3-like (*Dus3l*)	0	−1.394 (0.002)
Cerebellar degeneration-related 2 (*Cdr2*)	0.125	−1.433 (0.003)
ADP-ribosylation factor 4-like (*Arfl4*)	0.125	−1.591 (0.001)
Deoxyhypusine synthase (*Dhps*)	0.125	−1.179 (0.003)
Ephrin receptor B1 (*Ephb1*)	0.146	−1.892 (0.003)
Cell cycle-related kinase (*Ccrk*)	0.146	−1.334 (0.000)
Phosphofructokinase, liver (*Pfkl*)	0.146	−1.382 (0.008)
ELMO domain containing 2 (*Elmod2*)	0.146	−5.603 (0.001)
Interleukin 1 receptor-like 1 (*Il1rl1*)	0.184	−1.246 (0.007)
Transcription factor jun-D (*Jund1*)	0.184	−2.128 (0.004)
Bcl2-like 1 (*Bcl2l1*)	0.184	−1.775 (0.011)
Nuclear receptor interacting protein 2 (*Nrip2*)	0.184	−2.658 (0.011)
Similar to Deltex3 (*LOC100045005*)	0.184	−1.507 (0.004)
Stomatin-like 1 (*StomL1*)	0.184	−1.839 (0.001)
Coiled-coil domain containing 75 (*2310002B06Rik*)	0.184	−1.441 (0.005)
Scleraxis (*Scx*)	0.199	−2.580 (0.005)
Leucine-rich repeat-containing 1 (*Lrrc1*)	0.199	−1.527 (0.005)
Leucine-rich repeat-containing 45 (*Lrrc45*)	0.199	−1.289 (0.004)

Analysis performed using Significance Analysis for Microarrays (SAM). A False Discovery Rate (FDR) cut-off 0.2 was used.

Real-time PCR for the same genes was also performed on the C57BL/6 strain in order to determine whether the expression changes were occurring in the disease resistant strain of mice. The expression of *Spon2, Gdf5*, and *Ephb1* was found unchanged in *Cd151* ^−/−^ glomeruli of the C57BL/6 strain, suggesting that these expression changes are associated with the diseased phenotype. The down regulation of *Scx* and *Tspan32* genes however occurred in *Cd151* ^−/−^ glomeruli of both strains of mice indicating that it is an effect of *Cd151* absence regardless of glomerular disease susceptibility.

### Identification of differentially activated signalling pathways between FVB/N *Cd151*^+/+^ and *Cd151*^−/−^ glomeruli

To understand the signalling processes that were associated with the early pathological changes in the glomerulus, we performed Gene Set Enrichment Analysis (GSEA, Broad Institute, Cambridge, Massachusetts, US^23,24^). Gene sets that met the criteria of enrichment with *p* < 0.01 and FDR of 0.15 are shown in Table 2. In accordance with effacement of podocyte foot processes and loosening of their anchorage to the GBM, GSEA revealed that a large number of the differentially expressed genes were involved in the regulation of focal adhesions, the actin cytoskeleton and axon guidance. Interestingly, there was also a significant enrichment in pathways contributing to inflammation such as chemokine and T-cell receptor signalling pathways.

**Table 2:** KEGG gene sets differentially expressed between FVB/N *Cd151* ^−/−^ and *Cd151* ^+/+^ glomeruli.

KEGG pathway	Size	NES	Nominal *p*-val	FDR q-val
Peroxisome	69	1.99032	0.00000	0.00396
Cell cycle	108	−1.90468	0.00000	0.02657
Pancreatic cancer	65	−1.93288	0.00000	0.02905
Tryptophan metabolism	35	1.69974	0.00891	0.04498
Complement and coagulation cascades	61	1.63739	0.00293	0.06635
Chemokine signaling pathway	154	−1.77056	0.00000	0.07083
Systemic lupus erythematosus	82	−1.72188	0.00000	0.08722
Focal adhesion	167	−1.65367	0.00085	0.09668
Dilated cardiomyopathy	81	−1.63113	0.00384	0.10216
Regulation of actin cytoskeleton	182	−1.58601	0.00000	0.11907
T cell receptor signaling pathway	98	−1.58748	0.00081	0.12942
Renal cell carcinoma	64	−1.52747	0.00655	0.14551
Vascular smooth muscle contraction	96	−1.50814	0.00947	0.14636
Axon guidance	112	−1.49153	0.00894	0.15000

An FDR cut-off of 0.15 and *p*-value < 0.01 were used. Size: number of genes contributing to gene set enrichment. Abbreviations: NES, Nominal Enrichment Score; FDR, False Discovery Rate.

To further elucidate the pathological changes occurring with deletion of *Cd151* in the context of glomerular disease, the expression changes in FVB/N *Cd151* ^−/−^ glomeruli were analyzed with Ingenuity Pathway Analysis (IPA). Specifically analysing gene signatures associated with kidney function or disease identified 20 differentially expressed genes (Table 3, *p* < 0.05, fold change ≥ ±1.2). Interrogating IPA for pathways in which the differentially expressed genes were overrepresented revealed the genes were involved in neuronal development and axon guidance (*Gdnf, Adora2b and Htt*), inflammation (*Il-18, Tnfrsf1a, Tnfrsf22*), apoptosis (*Jun, Jund, Bcl2-l1*), and the turnover of basement membranes (*Xylt2, Loxl1, Timp1*). Contributing to the enrichment in the axon guidance signalling pathway, *Gdnf* (glial cell derived neurotrophic factor), a growth factor essential for both renal and neuronal development, was increased 4-fold in the FVB/N *Cd151* ^−/−^ diseased glomeruli. This upregulation was confirmed by immunofluorescence labelling which showed an increase in GDNF protein level in FVB/N *Cd151* ^−/−^ glomeruli, whilst no change was evident in C57BL/6 mice (supplementary Figure 1).

**Table 3:** IPA biomarker list of differentially expressed genes known to be involved in renal disease.

Gene name (*Symbol*)	Function	Fold change (*p*-value)
CD151 (Raph blood group) (*Cd151*)	Cell adhesion and integrin trafficking and/or function	Abolished (0)
Glial cell derived neurotrophic factor (*Gdnf*)	Neuronal cell survival	4.04 (0.006)
Protein kinase C, beta (*Prkcb*)	B cell activation, apoptosis induction, endothelial cell proliferation, regulation of neuronal function	−3.656 (0.025)
Tumour necrosis factor receptor superfamily, member 22 (*Tnfrsf22/Tnfrsf23*)	Cytokine receptor involved in inflammation	−3.361 (0.003)
Interleukin 18 (interferon-gamma-inducing factor) (*Il-18*)	Pro-inflammatory cytokine	2.727 (0.014)
Integrator complex subunit 6 (*Ints6*)	Involved in processing of RNA	−2.36 (0.042)
Jun D proto-oncogene (*Jund*)	(See Jun) also protects cells from p53-dependent senescence and apoptosis	−2.128 (0.004)
Jun proto-oncogene (*Jun*)	Involved in transcription regulation controlling differentiation, proliferation and apoptosis	−2.118 (0.026)
Haemoglobin, alpha 1 (*Hba1/Hba2*)	Forms haemoglobin (oxygen transport)	−2.034 (0.04)
BCL2-like 1 (*Bcl2l1*)	Apoptotic activator	−1.775 (0.011)
Huntingtin (*Htt*)	In neuronal cells promotes signalling, transport of materials, cytoskeletal anchorage and cell survival	1.738 (0.031)
Tissue inhibitor of metallopeptidase 1 (*Timp1*)	Inhibits matrix metalloproteinases	1.716 (0.009)
Lysyl oxidase-like 1 (*Loxl1*)	Essential in biogenesis of connective tissue as it catalyses the formation of crosslinks in collagens and elastins	−1.537 (0.016)
Tumour necrosis factor receptor superfamily, member 1 A (Tnfrsf1a)	Activates transcription factor Nuclear Factor -κβ (cell stress response regulator), mediator of apoptosis, regulator of inflammation	−1.405 (0.028)
Ceruloplasmin (ferroxidase) (*Cp*)	Iron and copper metabolism	1.381 (0.022)
Adenosine A2b receptor (*Adora2b*)	Stimulates adenylate cyclase activity, interacts with netrin 1 to promote axon elongation	−1.376 (0.022)
Xylosyltransferase II (*Xylt2*)	Biosynthesis of proteoglycans	−1.218 (0.02)
ERI1 exoribonuclease family member 3 (*Eri3*)	RNA degradation	−1.214 (0.02)
Phosphofructokinase, liver (*Pfkm*)	Enzymatic regulator of glycolysis	−1.382 (0.008)
Phosphofructokinase, muscle (*Pfkl*)	Enzymatic regulator of glycolysis	−1.393 (0.026)

The data was filtered before entry into IPA biomarker, selecting only genes with fold difference > ± 1.2 and *p*-value < 0.05.

### Induction of MMP-10 is associated with glomerular disease in FVB/N *Cd151*^−/−^ glomeruli

**Figure 2:**
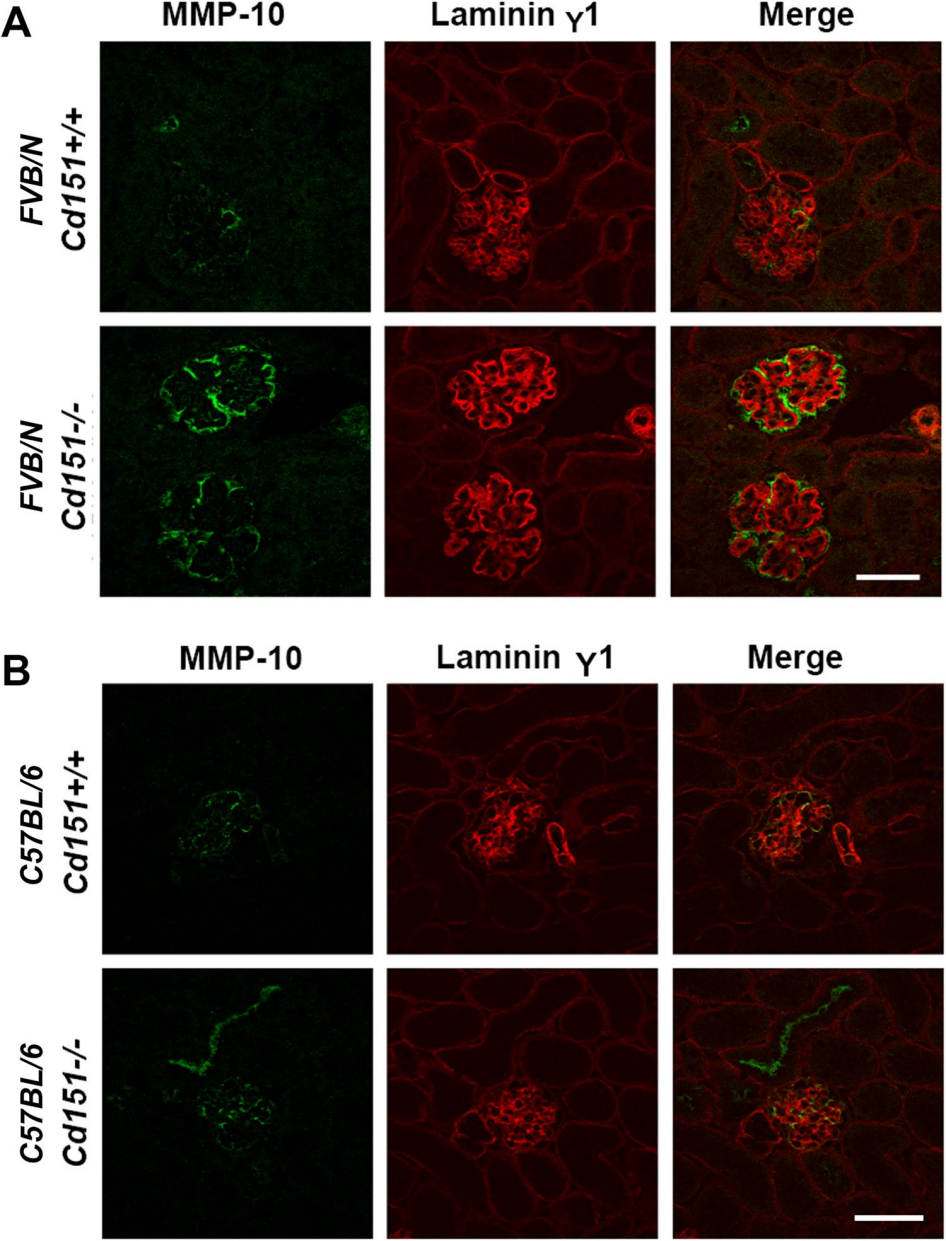
Localisation of MMP-10 in the glomeruli of 3-week-old FVB/N *Cd151* ^−/−^ mice. Dual immunofluorescence labelling and confocal analysis using an MMP-10 antibody (green) and a laminin γ1 antibody (red). (**A**) Strong fluorescence intensity is observed in the FVB/N *Cd151* ^−/−^ glomeruli as compared to very weak labelling in FVB/N *Cd151* ^+/+^ controls. In the FVB/N *Cd151* ^−/−^ glomeruli, the MMP-10 labelling mostly localises adjacent to the laminin γ1 staining, on the podocyte side of the GBM. (**B**) In contrast, only low MMP-10 labelling was detected in C57BL/6 kidneys, regardless of the *Cd151* ^+/+^ and *Cd151* ^−/−^ genotypes. Representative images are shown; n = 5 per mouse group; Original Magnification × 400. Bar: 50 μm.

We investigated the expression of genes encoding matrix metalloproteinases (MMPs) in our microarray results since MMPs are crucial in ECM homeostasis and turnover and they have been implicated in the pathology of many glomerular diseases including Alport syndrome^25–27^. Moreover, tetraspanins are known to regulate MMPs^25,28–31^. Whilst none of the *Mmp* genes appeared in the *p*-value filtered list of differentially expressed genes (Supplementary Tables S2 and S3, with p < 0.05), on examination of the unfiltered results *Mmp-10* was the only one showing a large fold change (x10.2) that was approaching significance (*p* = 0.08). We further assessed the expression of *Mmp-10* using real-time PCR, which confirmed that *Mmp-10* expression was significantly increased (by 26.9 fold) in FVB/N *Cd151* ^−/−^ compared to *Cd151* ^+/+^ glomeruli (*p* = 0.04) and not significantly different in C57BL/6 *Cd151* ^−/−^ glomeruli compared to C57BL/6 *Cd151* ^+/+^ controls (*p* = 0.19) (Fig. 1B). Further, immunofluorescence analysis correlated with transcript levels where MMP-10 protein levels were increased in the glomeruli of FVB/N *Cd151* ^−/−^ compared to FVB/N *Cd151* ^+/+^ mice (Fig. 2A). In addition, immunolabelling of MMP-10 in both C57BL/6 *Cd151* ^+/+^ and C57BL/6 *Cd151* ^−/−^ showed low fluorescence intensity, similar to that observed in FVB/N *Cd151* ^+/+^ glomeruli (Fig. 2A,B). Co-labelling with the basement membrane protein laminin γ1 demonstrated that the expression of MMP-10 was localised to the glomerular basement membrane adjacent to the glomerular epithelium (Fig. 2A).

**Figure 3:**
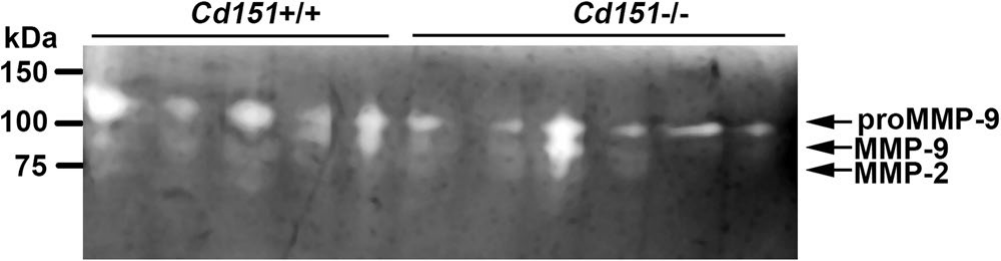
Gelatinase activity in 3-week-old FVB/N *Cd151* ^+/+^ and *Cd151* ^−/−^ mouse kidneys. Gelatinase activity in 3-week-old glomerular protein isolates from *Cd151* ^+/+^ and *Cd151* ^−/−^ FVB/N mice (n = 8 per group) was assessed using gelatin gel zymography. While a trend towards overall reduced activity in the *Cd151* ^−/−^ samples was noticeable on the gels, quantitation of band intensity revealed that there was no statistically significant difference between the 2 groups of mice (*p* = 0.41 for pro-MMP-9; *p* = 0.10 for MMP-9; *p* = 0.40 for MMP-2, by student’s t-test). Cropped gel image is displayed.

Altered expression of the gelatinases MMP-2 and MMP-9 has been associated with various glomerulopathies characterised by GBM defects, including diabetic nephropathy and Alport syndrome. In addition to regulation through gene expression changes, MMP activity is also modulated by other molecules such as TIMPs (Tissue Modulators of Matrix Metalloproteases). Despite *Mmp-2* and *Mmp-9* expression being unchanged in our microarray expression data, we took a closer look at their activity using gelatin gel zymography. According to the apparent molecular weights, activity of pro-MMP-9/MMP-9 was predominant in all glomerular samples whereas MMP-2 activity was negligible (Fig. 3). A trend towards reduced MMP-9 gelatinase activity was detected in the glomeruli of FVB/N *Cd151* ^−/−^ mice compared to FVB/N *Cd151* ^+/+^ but this change was not statistically significant (Fig. 3). Interestingly, *Timp1* expression was significantly upregulated by 1.7 fold in the microarray result when comparing FVB/N *Cd151* ^−/−^ to *Cd151* ^+/+^ glomeruli (Supplementary Table S2, p = 0.009).

### The extracellular matrix protein mindin appears as an early biomarker of GBM damage in FVB/N *Cd151*^−/−^ mice

As disruption of the GBM is a prominent feature in FVB/N *Cd151* ^−/−^ mice, we took a closer look at GBM components and modulators in the list of differentially expressed genes in the FVB/N *Cd151* ^−/−^ glomeruli. Firstly, expression of the laminin (*Lama5, Lamb1, Lamb2, Lamc1, Nidogen*) and collagen (*ColIVa4 and ColIVa5*) chains that we have previously shown to accumulate in the GBM^4^ were not significantly changed in expression (not shown), further suggesting protein accumulation rather than gene expression changes. Interestingly, the *Ntn4* gene, encoding the basement membrane protein netrin 4, was significantly downregulated (p = 0.04). Netrin 4 is part of a family of extracellular matrix proteins that serve as potent axon guidance molecules^32^. The decrease in *Ntn4* mRNA was confirmed by real-time PCR in *Cd151* ^−/−^ glomeruli of both FVB/N and C57BL/6 mice (Fig. 1B). However, the ~2 fold decreased *Ntn4* mRNA level did not translate into an obvious decrease in netrin 4 protein amount as observed by immunofluorescence in the glomeruli of *Cd151* ^+/+^ and *Cd151* ^−/−^ mice of both strains (Supplementary Figure 2). Instead, when examining the FVB/N *Cd151* ^−/−^ glomeruli, stronger netrin 4 labelling was observed in areas of thickened GBM (Supplementary Figure 2).

**Figure 4:**
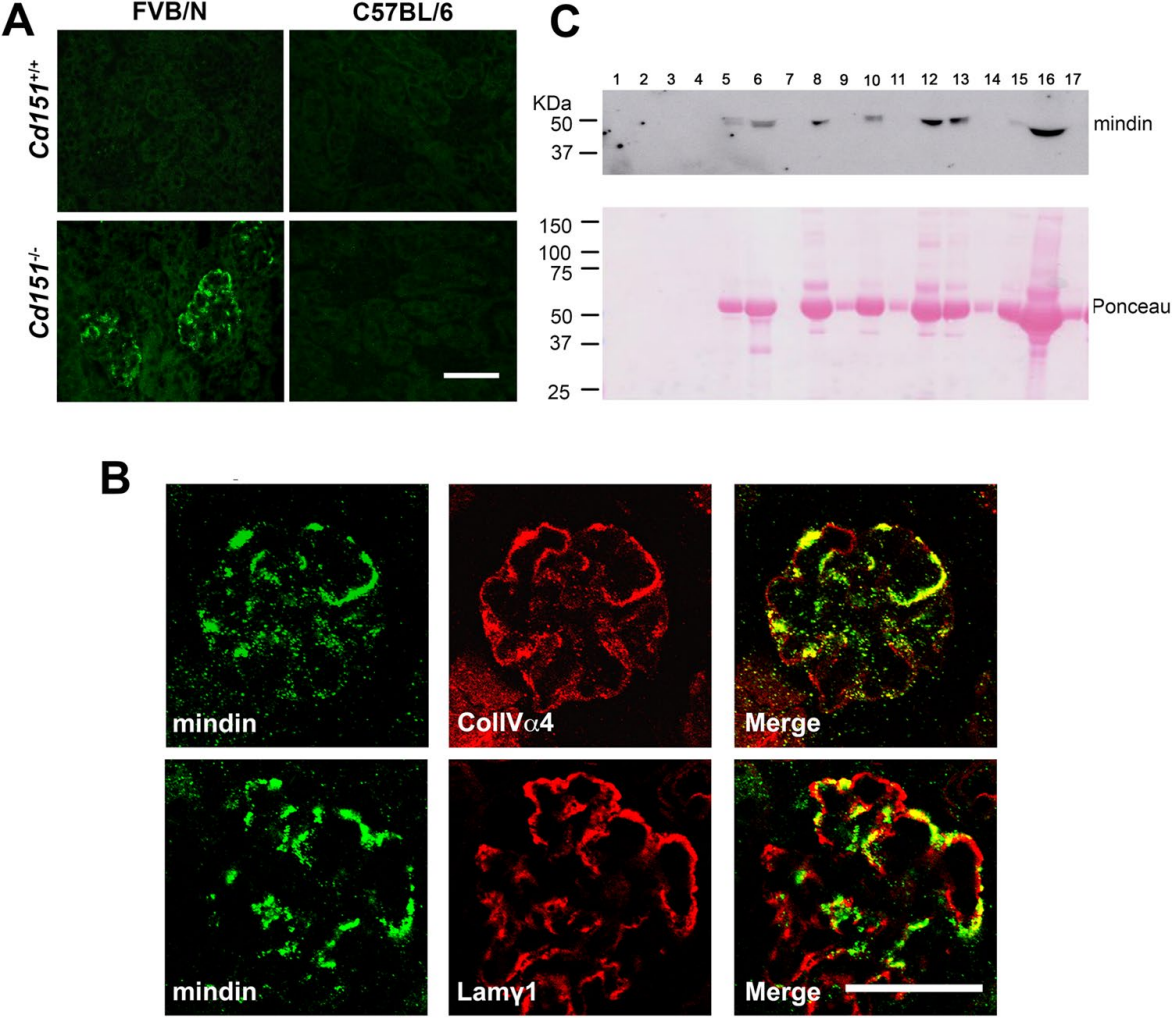
Induction of mindin in the diseased GBM of FVB/N *Cd151* ^−/−^ mice. (**A**) Representative immunofluorescent labelling of mindin in 3-week-old *Cd151* ^+/+^ and *Cd151* ^−/−^ kidneys on the FVB/N and C57BL/6 backgrounds. Strong mindin immunolabelling was observed specifically in the glomeruli of FVB/N *Cd151* ^−/−^ mice, whereas the staining was negative in age-matched kidneys from FVB/N *Cd151* ^+/+^ and C57BL/6 *Cd151* ^+/+^ and *Cd151* ^−/−^ mice (n = 8 per group). Original magnification × 400. Bar 50 μm. (**B**) Dual immunofluorescence labelling and confocal analysis of mindin (green) and the GBM components collagen IV α4 chain (Top, red) or laminin γ1 (Bottom, red) in 3-week-old FVB/N *Cd151* ^−/−^ kidneys. One representative glomerulus is shown for each co-labelling (n = 3 mice). Mindin colocalises substantially with both collagen IV α4 and laminin γ1 in the GBM. Original magnification × 800. Bar: 50 μm. (**C**) Detection of mindin in the urine of 3-week-old FVB/N *Cd151* ^+/+^ and *Cd151* ^−/−^ mice by western blot analysis (top) and Ponceau staining of the same blot (bottom). Lane 1 to 4 and 7: healthy FVB/N *Cd151* ^+/+^ sample; lanes 5, 6 and 8 to 17 show the variability in FVB/N *Cd151* ^−/−^ samples with 8 out of 12 having detectable amounts of mindin. n = 5 for *Cd151* ^+/+^ and n = 12 for *Cd151* ^−/−^.

Of particular interest for its function in cell adhesion and activation of the immune system^33–37^, *Spon2* (encoding the extracellular matrix protein mindin), was identified using SAM as the most significantly upregulated gene in the expression dataset and confirmed by real-time PCR (Fig. 1A, >10 fold). Immunofluorescent labelling of 3-week-old kidneys showed specific expression and accumulation of mindin in the glomeruli of 3-week-old FVB/N *Cd151* ^−/−^ mice (Fig. 4A), in accordance with the increased *Spon2* expression. FVB/N *Cd151* ^+/+^ as well as C57BL/6 *Cd151* ^−/−^ and *Cd151* ^+/+^ kidney sections did not show any mindin immunolabelling, suggesting that the low levels of *Spon2* mRNA detected in glomerular extracts of these mice may not be physiologically relevant. In order to better define the localisation of mindin in the FVB/N *Cd151* ^−/−^ diseased glomeruli, we performed double immunofluorescent labelling and confocal analysis in FVB/N *Cd151* ^−/−^ kidneys with antibodies against the GBM proteins laminin γ1 and COLIVα4. The dual labelling showed overlapping mindin staining with both the laminin γ1 and COLIVα4 labelling, demonstrating that mindin accumulates in the GBM of the diseased FVB/N *Cd151* ^−/−^ glomeruli (Fig. 4B). Moreover, mindin staining was stronger in areas of thickened GBM suggesting that it contributes to the thickened and disorganised GBM. In order to determine if the induction of mindin could have a direct involvement in the disorganisation of the GBM and therefore the development of glomerular disease in these mice, we performed mindin immunolabelling on kidney sections at earlier time points. Mindin immunolabelling of kidneys at 5 days of age or just before birth (18.5 dpc) gave negative results in C57BL/6 and FVB/N mice of both *Cd151* ^−/−^ and *Cd151* ^+/+^ genotypes and no difference was observed in total kidney *Spon2* mRNA levels at 5 days of age (data not shown). Altogether these results suggest that mindin is a relatively early marker of glomerular injury in FVB/N *Cd151* ^−/−^ mice but is not involved in the initial events resulting in glomerular disease. Moreover, we tested the urine of the *Cd151* ^−/−^ mice by western blotting using anti-mindin antibodies. Mindin was detected in 8 out of 12 (>65%) 3-week-old FVB/N *Cd151* ^−/−^ mice tested and none of the controls (Fig. 4C), further suggesting its potential as a useful biomarker of glomerular pathology. Ponceau staining revealed that the samples positive for mindin at this age were also the ones with substantial levels of protein in the urine spot collection, whereas the mice with low level of proteinuria tested negative (Fig. 4C). Kidney pathology was assessed with H&E staining and showed no clear association with the severity of glomerular lesions at this early stage of disease progression (not shown).

## Discussion

Morphological changes of the GBM occur in a variety of glomerulopathies whether they are caused by an intrinsic defect of the kidney filter such as the defective collagen IV network in Alport syndrome or by a systemic disease such as diabetes in the case of diabetic nephropathy. The *Cd151* knock-out mouse model represents a valuable tool for the investigation of the molecular mechanisms involved in glomerular diseases associated with GBM defects, that could lead to identification of biomarkers and/or therapeutic targets for prognosis and treatment of a large subset of glomerular diseases.

In this study, we characterised glomerular gene expression changes associated with the early stages of glomerular disease in 3-week-old FVB/N *Cd151* ^−/−^ mice. Systematic analysis of the microarray expression data using IPA and GSEA revealed an enrichment of gene expression changes involved in specific pathways. Differential expression was identified in genes previously associated with renal function and disease that seemed to cluster mostly into four functional groups: apoptosis, basement membrane regulation, axon guidance, and inflammation. The expression changes in genes involved in basement membrane regulation is in accordance with the main ultrastructural defect observed in FVB/N *Cd151* ^−/−^ kidneys that is the thickening and splitting of the GBM. The change in expression of apoptosis/cell survival genes and activation of inflammatory pathways, in addition to loosening of podocyte anchorage to the GBM, may reflect the mechanisms involved in podocyte loss that is significant in FVB/N *Cd151* ^−/−^ glomeruli at this age^4^. This hypothesis is supported by several reports that show albuminuria can trigger an inflammatory response in podocytes and elicits apoptotic cell death^38^. Moreover, GSEA also identified an enrichment in large gene sets associated with inflammation related signalling (chemokine and T-cell receptor pathways), as well as the axon guidance pathway, focal adhesions and the actin cytoskeleton. The axon guidance, chemokine receptor and focal adhesion pathways all affect the downstream regulation of the actin cytoskeleton and therefore any or all of these could be leading to the widespread gene expression changes in the actin cytoskeleton network. Inspection of the key transcript changes contributing to enrichment in the axon guidance signalling pathway indeed suggested an overlap with deregulation of the actin cytoskeleton. The actin cytoskeleton is known to be crucial for the maintenance of the structure of the actin-rich podocyte foot processes. Disruption of the podocyte actin cytoskeleton, signified by effacement of podocyte foot processes, is a major feature of a large number of glomerular pathologies, including glomerular disease associated with *CD151* mutations^4,5^. Furthermore, given that CD151 strengthens integrin adhesions, its deletion must not only loosen these sites of anchorage but also directly affect integrin signalling to the actin cytoskeleton. Podocyte foot processes are known to require guidance cues in order to maintain their tight association and function inside the glomerular filtration barrier under normal conditions and stress related to glomerular damage^39–41^. Several molecules involved in axon guidance signalling have been reported previously to affect podocyte signalling to and regulation of the GBM^41,42^. Reciprocally, loosening of the podocyte integrin-GBM adhesion as a result of *Cd151* deletion might result in a response of the podocyte, through axon guidance cues, in an attempt to compensate and maintain proper coverage of the GBM. For example, the gene encoding the tyrosine kinase receptor EphB1 was differentially expressed in *Cd151* ^−/−^ glomeruli. EphB1 is involved in axon guidance as well as integrin-based cell adhesion^43^ and has also been involved in glomerular development^44,45^. These findings further support emerging evidence that axonal dendrite and podocyte foot process formation and maintenance are governed by overlapping cues^39–42,46–48^.

Intriguingly the expression of *Tspan32* (also known as *Tssc6*), which encodes another tetraspanin protein, was abolished in FVB/N *Cd151* ^−/−^ glomeruli. The mechanism involved remains to be determined. *Tspan32* expression was equally abolished in C57BL/6 *Cd151* ^−/−^ mice, which have healthy kidneys and characterisation of *Tspan32* ^−/−^ mice (on a mixed C57BL/6-SPRET/Ei genetic background) showed no overt kidney phenotype^49^, which suggests it is not essential for glomerular function.

This study also revealed alteration in expression of several basement membrane regulators that likely contribute to the characteristic GBM ultrastructural damage seen early in the FVB/N *Cd151* ^−/−^ kidneys. Expression of MMPs occurs with disease progression in Alport syndrome and MMP-2, MMP-9, and MMP-12 have been linked to GBM alterations^26,27^. CD151 is known to influence both the expression and activation of MMPs. For example, CD151 regulates the expression of MMP-2,-7 and -9^30,31^ and activation of MMP-7^28^ and MMP-2^29^. Furthermore, Delimont *et al*. have reported increased expression of *Mmp-10* and *Mmp-12* in the glomeruli of 5-week-old FVB/N *Cd151* ^−/−^ mice^25^. When the current study began, the expression and activity of MMPs in the absence of CD151 had not been studied in the kidney. Surprisingly, amongst all MMPs, the expression data suggested differential expression of the stromelysin encoding gene *Mmp-10* only. Substantial MMP-10 protein levels were evident specifically in FVB/N *Cd151* ^−/−^ glomeruli along the podocyte side of the GBM, suggesting that it is produced by the podocytes. In contrast to Delimont *et al*., we did not observe *Mmp-12* induction alongside *Mmp-10* in our model. This difference could be explained by the age difference of the experimental animals used, whereby our study was performed at 3 weeks of age and the other at 5 weeks^25^. Moreover, in our model, the genes encoding the gelatinases *Mmp-2* and *Mmp-9* were not differentially expressed at 3 weeks of age and further investigation of their activity did not reveal any significant difference. However, the trend towards a non-significant decrease in gelatinase activity in the FVB/N *Cd151* ^−/−^ glomeruli compared to FVB/N *Cd151* ^+/+^ suggests that there could be significant alterations in the activity of MMP-2 and MMP-9 at later stages of disease but this remains to be tested. In glomerular diseases, such as Alport syndrome and diabetic nephropathy, the pathological changes in the GBM result from both altered matrix component synthesis and defective MMP degradation^50,51^ and our results suggest a similar mechanism is occurring in the absence of CD151. The significant increase in *Timp-1* expression in FVB/N *Cd151* ^−/−^ glomeruli could be a response from the podocytes to compensate for the increase in MMP-10. Indeed, TIMP-1 binds to and inhibits many of the MMPs known to be regulated by CD151, including MMP-2, -7 and -9^52^ and also inhibits MMP-10. However, given the significantly increased MMP-10 protein levels and the small increase in *Timp-1* expression, it is tempting to speculate that MMP-10 activity is increased in the diseased *Cd151* ^−/−^ glomeruli. The activity of stromelysins (MMP-3 and MMP-10) can be assessed on casein substrate, but they have typically lower catalytic activity than other MMPs such as gelatinases. In addition to its MMP inhibitory function, TIMP-1 has been implicated in epithelial cell turnover and survival^53^, repair^54^ and polarisation^55^. TIMP-1 also regulates cell survival and polarisation through modulation of tetraspanin/integrin signalling complexes such as those present on podocyte foot processes^55^. While this remains to be further tested, increased *Timp1* expression could therefore also contribute to the podocyte ultrastructural changes occurring with disease onset.

The large induction of mindin expression in the diseased GBM and its presence in the majority of urine samples from FVB/N *Cd151* ^−/−^ mice suggests that this protein could have some potential use as a biomarker across a range of glomerular diseases associated with ultrastructural GBM damage. Indeed, mindin has been reported previously as an early biomarker of diabetic nephropathy^56^ and our findings show that it is also induced in the GBM in the Alport-like glomerular disease model of FVB/N *Cd151* ^−/−^ mice. While the presence of mindin correlated with the amount of protein in the urine, there was no clear association with the glomerular pathological features of the corresponding 3-week-old *Cd151* ^−/−^ kidneys, which are relatively mild at this early stage of disease progression, as reported previously^4^. Further investigations will be required to determine the exact role of mindin in disease progression, whether it is found in the GBM and urine of patients affected with Alport syndrome and other glomerulopathies. Interestingly mindin directly binds integrins, modulates their activity^35^ and has been shown to play a role in cell-basement membrane adhesion^33^. Perturbation of integrin signalling and the increasing mechanical strain inflicted onto the podocytes upon *Cd151* deletion could lead to *Spon2* induction as an attempt to strengthen the hold of the foot processes onto the disturbed GBM. Moreover, in the innate immune system, mindin plays a role in activation of macrophages, mast cells and T-cells, production and activation of pro-inflammatory cytokines, and recruitment of neutrophils, macrophages and eosinophils^33–37^. In the context of the glomerular filtration barrier, considering the dual role of mindin in cell/matrix adhesion and in inflammation, it is tempting to speculate, similarly to Murakochi and colleagues^56^, that mindin plays an important role in glomerular disease progression, at the interface between the podocyte maladaptive signalling changes to the actin cytoskeleton and the activation of pro-inflammatory pathways, causing further podocyte damage and ultimately leading to progression of the disease, podocyte loss, and glomerulosclerosis. These findings suggest a role for mindin as an early biomarker of the progression of glomerular diseases associated with GBM ultrastructural defect and this hypothesis will have to be tested in humans. It will also be important to determine in future experiments whether podocyte/glomerular deletion of *Spon2* has the ability to slow down glomerular disease progression.

## Methods

### Animals

The use of animals was approved by the University of Newcastle’s (Australia) Animal Care and Ethics Committee. All experimental procedures were performed in accordance with the New South Wales Animal Research Act, New South Wales Animal Research Regulation, and the Australian code for the care and use of animals for scientific purposes. Genotyping was performed as previously described^6^. Only male mice were used in the microarray experiment and validation experiments to avoid variations due to gender.

### Isolation of Glomeruli

Glomeruli were isolated from mouse kidneys at 3 to 4 weeks of age using a technique described previously by Takemoto and colleagues^57^. Briefly, a solution containing 8 × 10^7^ tosyl-inactivated Dynabeads (Dynabeads M450, Dynal M450, Invitrogen, Carlsbad, CA, USA) in 40 mL of HBSS (Hank’s Buffered Salt Solution, Invitrogen) was perfused through the heart of deeply anaesthetised mice. The freshly isolated kidneys were minced and gently digested with a solution containing 1 mg/mL collagenase A (Roche, West Sussex, UK) and 100 U/mL DNase 1 (Invitrogen) at 37 °C for 20 min. The homogenates were washed on a magnetic rack (Dynal® MPC-S, Invitrogen) until a purity of minimum 95% glomeruli per sample was achieved.

### RNA extraction

Total RNA was extracted from isolated glomeruli using the RNeasy Micro kit, as per the manufacturer’s instructions (Qiagen, Hilden, Germany).

### Microarray analysis

Glomerular RNA samples of FVB/N *Cd151* ^−/−^ and FVB/N *Cd151* ^+/+^ 3-week-old mice (n = 4 per group) were prepared in accordance with Illumina recommended protocols. Briefly, RNA from each sample was amplified and labelled using the Illumina TotalPrep RNA Amplification Kit. Concentrations of cRNA were determined with the Quant-iT RiboGreen RNA Assay Kit (Invitrogen) and 750ng of biotin-labelled cRNA was hybridised (17 h) to MouseRef-8 v1.1 Expression BeadChip (Illumina, San Diego, CA, USA). The hybridised biotinylated cRNA was detected with Cy3-streptavidin and quantitated using the Illumina BeadArray Reader.

Microarray data was subject to quality control testing in Genome Studio® (Illumina) ensuring that no error was introduced between array chips and that intensity values were not affected by artefacts such as poor hybridisation. Cubic spline normalised data was filtered for detection *P*-value ([n > 0] ≤ 0.05) to remove genes that were not detected in any samples. The filtered data set was analysed using SAM, with 3000 permutations and 20% FDR to identify only the most highly significant gene expression changes. Pathway analysis was performed using a gene list including all genes identified as differentially expressed at *p* < 0.05 for GSEA or *p* < 0.05 plus fold change ≥ ±1.2 for IPA. Significance was determined for GSEA results at *p* < 0.05 and FDR q-value < 0.1 with the gene set permutation setting (as recommended when n < 8) and the KEGG pathway database provided by GSEA as the reference. IPA was used to identify kidney disease signature genes within the up-regulated and down-regulated genes in *Cd151* ^−/−^ glomeruli.

### Real-time PCR

Glomerular RNA (500ng) samples from 4 each of FVB/N *Cd151* ^−/−^, FVB/N *Cd151* ^+/+^, C57BL/6 *Cd151* ^−/−^ and C57BL/6 *Cd151* ^+/+^ 3-week-old mice were reverse transcribed with the Superscript III First Strand Synthesis Kit (Invitrogen) according to the manufacturer’s instructions. The geometric mean of 3 housekeeping genes, Glyceraldehyde 3-phosphate dehydrogenase (*Gapdh*), 18S ribosomal sub-unit (18S) and Beta-glucuronidase (*Gusb*) was used to normalise the real-time PCR data. Each reaction (12.5 μL) consisted of 1 × Power SYBR® Green PCR Master Mix (Applied Biosystems), 2–3 nM of each forward and reverse primer and diluted cDNA (See Table S1 for primer sequences). After an initial enzyme activation step for 10 min at 95 °C, 40 cycles of 15 s at 95 °C and 45 s at 60 °C (or 62 °C for *Scx*) were completed on an ABI PRISM 7500 RT-PCR System (Applied Biosystems). For each gene, all samples were run in triplicates and the experiment was repeated twice.

Student’s t-tests were used to compare the cycle thresholds of housekeeping genes between *Cd151* ^−/−^ and *Cd151* ^+/+^ in both FVB/N and C57BL/6 strains. An F-test was used to compare the distribution across all samples between the three house-keeping genes. Statistical significance between *Cd151* ^−/−^ and *Cd151* ^+/+^ ∆Ct’s were determined using student’s t-tests.

### Immunofluorescence labelling

Immunofluorescence labelling were performed on 3 μm frozen sections as previously described^4^. Primary antibodies and dilutions used for immunofluorescent labellings were rabbit anti-GDNF at 1:100 (NBP1-45595, Novus Biologicals, CO, USA), rabbit anti-MMP10 at 1:100 (H-300, Santa Cruz Biotechnologies, Santa Cruz, CA, USA), rabbit anti-mindin at 1:50 (H131, Santa Cruz Biotechnologies, Santa Cruz, CA, USA), rabbit anti-netrin 4 at 1:500 (KR92, generously provided by Dr Manuel Koch, Cologne, Germany^32^), and rat anti-laminin γ1 at 1:1000 (MAB1914, Chemicon, Temecula, CA, USA). Appropriate rabbit and rat isotype IgG controls were included during optimization. Donkey anti-rabbit AlexaFluor 488 and goat anti-rat AlexaFluor 594 secondary antibodies (Molecular Probes, Invitrogen) were used at 1:300 dilutions. Sections were mounted with Prolong® Gold Antifade Reagent (Invitrogen) and images captured using the Olympus BX51 (Olympus, Shinjuku, Tokyo, Japan) and the Fluoview FV1000 confocal microscope (Olympus).

### Gelatin gel zymography

Protein was precipitated from homogenised glomerular isolates in 4 volumes of ice-cold acetone and incubated on ice for 30 min, pellets were washed once in 100 μL ice cold ethanol and then freeze dried and stored at −80 °C. Glomerular protein samples from 8 of each FVB/N *Cd151* ^−/−^ and *Cd151* ^+/+^ 3-week-old mice were normalised for concentration using the microBCA protein assay kit (Pierce Biotechnology, IL, USA). Proteins (20 μg) were electrophoresed on gelatin zymogram gels (Novex, Invitrogen) then renatured in 1X renaturing buffer for 30 min and after equilibration, incubated in 1X developing buffer at 37 °C overnight. Gels were then silver-stained for band detection and imaged using the Fujifilm LAS-4000 Imaging System (GE Healthcare, Buckinghamshire, UK). Quantification of bands was performed with densitometry analysis of inverted images in ImageJ and the results were compared using Student’s t-test.

### Western blotting

Urine samples (2 μL) were processed by SDS polyacrylamide gel electrophoresis and transferred onto nitrocellulose membrane. Briefly, after blocking in 5% skim milk powder for 45 min, membranes were probed with a goat anti-mindin antibody at 1:50 (sc-49049, Santa Cruz Biotechnologies, Santa Cruz, CA, USA) at 4 ^o^C overnight, followed by anti-goat horseradish peroxidase conjugated antibody 1:5000 (BioRad, California, USA) for 1 h at room temperature. Membranes were then visualized using enhanced chemiluminescence system ECL PLUS (ThermoFisher Scientific, Waltham, MA, USA) and imaged using the Fujifilm LAS-4000 Imaging System.

### Data Availability

All data generated or analysed during this study are included in this published article (and its Supplementary Information files) and the gene expression datasets have been deposited to the GEO repository with accession number GSE104624.

## Electronic supplementary material

Supplementary data

## References

[1] Kruegel J, Rubel D, Gross O (2013). *Alport syndrome–insights from basic and clinical research*. Nat Rev Nephrol.

[2] Marshall CB (2016). Rethinking glomerular basement membrane thickening in diabetic nephropathy: adaptive or pathogenic?. Am J Physiol Renal Physiol.

[3] Karamatic Crew V (2004). CD151, the first member of the tetraspanin (TM4) superfamily detected on erythrocytes, is essential for the correct assembly of human basement membranes in kidney and skin. Blood.

[4] Baleato RM, Guthrie PL, Gubler MC, Ashman LK, Roselli S (2008). Deletion of CD151 results in a strain-dependent glomerular disease due to severe alterations of the glomerular basement membrane. Am J Pathol.

[5] Sachs N (2006). Kidney failure in mice lacking the tetraspanin CD151. J Cell Biol.

[6] Wright MD (2004). Characterization of mice lacking the tetraspanin superfamily member CD151. Mol Cell Biol.

[7] Fitter S, Tetaz TJ, Berndt MC, Ashman LK (1995). Molecular cloning of cDNA encoding a novel platelet-endothelial cell tetra-span antigen, PETA-3. Blood.

[8] Hemler ME (2003). Tetraspanin proteins mediate cellular penetration, invasion, and fusion events and define a novel type of membrane microdomain. Annu Rev Cell Dev Biol.

[9] Hemler ME, Mannion BA, Berditchevski F (1996). Association of TM4SF proteins with integrins: relevance to cancer. Biochim Biophys Acta.

[10] Yanez-Mo M (1998). Regulation of endothelial cell motility by complexes of tetraspan molecules CD81/TAPA-1 and CD151/PETA-3 with alpha3 beta1 integrin localized at endothelial lateral junctions. J Cell Biol.

[11] Yauch RL, Berditchevski F, Harler MB, Reichner J, Hemler ME (1998). Highly stoichiometric, stable, and specific association of integrin alpha3beta1 with CD151 provides a major link to phosphatidylinositol 4-kinase, and may regulate cell migration. Mol Biol Cell.

[12] Sincock PM (1999). PETA-3/CD151, a member of the transmembrane 4 superfamily, is localised to the plasma membrane and endocytic system of endothelial cells, associates with multiple integrins and modulates cell function. J Cell Sci.

[13] Serru V (1999). Selective tetraspan-integrin complexes (CD81/alpha4beta1, CD151/alpha3beta1, CD151/alpha6beta1) under conditions disrupting tetraspan interactions. Biochem J.

[14] Yauch RL, Kazarov AR, Desai B, Lee RT, Hemler ME (2000). Direct extracellular contact between integrin alpha(3)beta(1) and TM4SF protein CD151. J Biol Chem.

[15] Sterk LM (2002). Association of the tetraspanin CD151 with the laminin-binding integrins alpha3beta1, alpha6beta1, alpha6beta4 and alpha7beta1 in cells in culture and *in vivo*. J Cell Sci.

[16] Winterwood NE, Varzavand A, Meland MN, Ashman LK, Stipp CS (2006). A critical role for tetraspanin CD151 in alpha3beta1 and alpha6beta4 integrin-dependent tumor cell functions on laminin-5. Mol Biol Cell.

[17] Sincock PM, Mayrhofer G, Ashman LK (1997). Localization of the transmembrane 4 superfamily (TM4SF) member PETA-3 (CD151) in normal human tissues: comparison with CD9, CD63, and alpha5beta1 integrin. J Histochem Cytochem.

[18] Sachs, N. *et al*. Blood pressure influences end-stage renal disease of Cd151 knockout mice. *J Clin Invest* **122**, 348–358, 10.1172/JCI58878. 10.1172/JCI58878 PMC3248294 22201679

[19] Tusher VG, Tibshirani R, Chu G (2001). Significance analysis of microarrays applied to the ionizing radiation response. Proc Natl Acad Sci USA.

[20] Meehan DT (2009). Biomechanical strain causes maladaptive gene regulation, contributing to Alport glomerular disease. Kidney Int.

[21] Levay AK (2008). Scleraxis is required for cell lineage differentiation and extracellular matrix remodeling during murine heart valve formation *in vivo*. Circ Res.

[22] Li X, Leo BM, Beck G, Balian G, Anderson GD (2004). Collagen and proteoglycan abnormalities in the GDF-5-deficient mice and molecular changes when treating disk cells with recombinant growth factor. Spine (Phila Pa 1976).

[23] Mootha VK (2003). Identification of a gene causing human cytochrome c oxidase deficiency by integrative genomics. Proc Natl Acad Sci USA.

[24] Subramanian A (2005). Gene set enrichment analysis: a knowledge-based approach for interpreting genome-wide expression profiles. Proc Natl Acad Sci USA.

[25] Delimont D (2014). Laminin alpha2-mediated focal adhesion kinase activation triggers Alport glomerular pathogenesis. PLoS One.

[26] Rao VH (2006). Role for macrophage metalloelastase in glomerular basement membrane damage associated with alport syndrome. Am J Pathol.

[27] Zeisberg M (2006). Stage-specific action of matrix metalloproteinases influences progressive hereditary kidney disease. PLoS Med.

[28] Fujita Y (2006). Tetraspanin CD151 is expressed in osteoarthritic cartilage and is involved in pericellular activation of pro-matrix metalloproteinase 7 in osteoarthritic chondrocytes. Arthritis Rheum.

[29] Yanez-Mo M (2008). MT1-MMP collagenolytic activity is regulated through association with tetraspanin CD151 in primary endothelial cells. Blood.

[30] Hong IK (2006). Homophilic interactions of Tetraspanin CD151 up-regulate motility and matrix metalloproteinase-9 expression of human melanoma cells through adhesion-dependent c-Jun activation signaling pathways. J Biol Chem.

[31] Hasegawa M (2007). CD151 dynamics in carcinoma-stroma interaction: integrin expression, adhesion strength and proteolytic activity. Lab Invest.

[32] Schneiders FI (2007). Binding of netrin-4 to laminin short arms regulates basement membrane assembly. J Biol Chem.

[33] Li Y (2009). Structure of the F-spondin domain of mindin, an integrin ligand and pattern recognition molecule. EMBO J.

[34] He YW (2004). The extracellular matrix protein mindin is a pattern-recognition molecule for microbial pathogens. Nat Immunol.

[35] Jia W, Li H, He YW (2005). The extracellular matrix protein mindin serves as an integrin ligand and is critical for inflammatory cell recruitment. Blood.

[36] Jia W, Li H, He YW (2008). Pattern recognition molecule mindin promotes intranasal clearance of influenza viruses. J Immunol.

[37] Guleng B, Lian YM, Ren JL (2010). Mindin is upregulated during colitis and may activate NF-kappaB in a TLR-9 mediated manner. World J Gastroenterol.

[38] Okamura K (2013). Endocytosis of albumin by podocytes elicits an inflammatory response and induces apoptotic cell death. PLoS One.

[39] Villegas G, Tufro A (2002). Ontogeny of semaphorins 3A and 3F and their receptors neuropilins 1 and 2 in the kidney. Mech Dev.

[40] Guan F, Villegas G, Teichman J, Mundel P, Tufro A (2006). Autocrine class 3 semaphorin system regulates slit diaphragm proteins and podocyte survival. Kidney Int.

[41] Reidy KJ (2009). Semaphorin3a regulates endothelial cell number and podocyte differentiation during glomerular development. Development.

[42] Fan X (2012). Inhibitory effects of Robo2 on nephrin: a crosstalk between positive and negative signals regulating podocyte structure. Cell Rep.

[43] Huynh-Do U (1999). Surface densities of ephrin-B1 determine EphB1-coupled activation of cell attachment through alphavbeta3 and alpha5beta1 integrins. EMBO J.

[44] Daniel TO (1996). ELK and LERK-2 in developing kidney and microvascular endothelial assembly. Kidney Int Suppl.

[45] Stein E (1998). Eph receptors discriminate specific ligand oligomers to determine alternative signaling complexes, attachment, and assembly responses. Genes Dev.

[46] Gerke P (2006). Neuronal expression and interaction with the synaptic protein CASK suggest a role for Neph1 and Neph2 in synaptogenesis. J Comp Neurol.

[47] Lefevre GM, Patel SR, Kim D, Tessarollo L, Dressler GR (2010). Altering a histone H3K4 methylation pathway in glomerular podocytes promotes a chronic disease phenotype. PLoS Genet.

[48] Brunskill EW, Georgas K, Rumballe B, Little MH, Potter SS (2011). Defining the molecular character of the developing and adult kidney podocyte. PLoS One.

[49] Nicholson RH (2000). Phemx, a novel mouse gene expressed in hematopoietic cells maps to the imprinted cluster on distal chromosome 7. Genomics.

[50] Ronco P, Lelongt B, Piedagnel R, Chatziantoniou C (2007). Matrix metalloproteinases in kidney disease progression and repair: a case of flipping the coin. Semin Nephrol.

[51] Thrailkill KM, Clay Bunn R, Fowlkes JL (2009). Matrix metalloproteinases: their potential role in the pathogenesis of diabetic nephropathy. Endocrine.

[52] Will H, Atkinson SJ, Butler GS, Smith B, Murphy G (1996). The soluble catalytic domain of membrane type 1 matrix metalloproteinase cleaves the propeptide of progelatinase A and initiates autoproteolytic activation. Regulation by TIMP-2 and TIMP-3. J Biol Chem.

[53] Guedez L, Courtemanch L, Stetler-Stevenson M (1998). Tissue inhibitor of metalloproteinase (TIMP)-1 induces differentiation and an antiapoptotic phenotype in germinal center B cells. Blood.

[54] Chen P (2008). Tissue inhibitor of metalloproteinase-1 moderates airway re-epithelialization by regulating matrilysin activity. Am J Pathol.

[55] Jung KK, Liu XW, Chirco R, Fridman R, Kim HR (2006). Identification of CD63 as a tissue inhibitor of metalloproteinase-1 interacting cell surface protein. EMBO J.

[56] Murakoshi M (2011). Mindin: a novel marker for podocyte injury in diabetic nephropathy. Nephrol Dial Transplant.

[57] Takemoto M (2002). A new method for large scale isolation of kidney glomeruli from mice. Am J Pathol.

