# Supplementary material for: Characterization of the early molecular changes in the glomeruli of Cd151−/− mice highlights induction of mindin and MMP-10
Source: Sci Rep. 2017 Nov 22;7:15987. doi: 10.1038/s41598-017-15993-3 (PMC5700190; doi:10.1038/s41598-017-15993-3)
Supplement: Supplementary file 1 — Supplementary data [file 41598_2017_15993_MOESM1_ESM.pdf]

Characterization of the early molecular changes in the glomeruli of *Cd151*$^{-/-}$ mice highlights induction of mindin and MMP-10

Crystal Naudin[1, 2, 3], Brian Smith[4], Danielle Bond[1,2], Matt Dun[1, 2], Rodney J. Scott[1, 2, 5], Leonie K. Ashman[1,2], Judith Weidenhofer [1, 2*] and Séverine Roselli[1,2*]

* These two authors contributed equally

1 School of Biomedical Sciences and Pharmacy, Faculty of Health and Medicine, University of Newcastle, New South Wales, Australia

2 Hunter Medical Research Institute, New Lambton, New South Wales, Australia

3 Emory University, Atlanta, Georgia, USA

4 School of Mathematics and Physical Sciences, University of Newcastle, Newcastle, New South Wales, Australia

5 Hunter Area Pathology Service, John Hunter Hospital, New Lambton, New South Wales, Australia

Supplementary Data

Supplementary Table S1. Real-time PCR primers

Gene ID	Accession	Forward primer (5’- 3’)	Reverse primer (5’- 3’)
18s	NR_003278.3	ATGCGGCGGCGTTATTCCCA	AGAACGGCCATGCACCACCA
Gapdh	NM_008084	CTGGAGAAACCTGCCAAGTA	CTGATTCATTGTCATACCAGG
Gusb	NM_010368	CTGCTCTGAAACCCGCCGCA	GGGCCCCCAGGTCTGCATCA
Spon2	NM_133903	ACCTTCGTGCGGCTACAGCA	ATCTGGGGCACACTCGGCCT
Tspan32	NM_001128080	GCTGCTGGGCCTCTCTATGGC	CCCCGCTAGGCTGATCCCCA
1810014F10Rik	NM_026928.2	AACTTCCCTACCTCCTCCATCTGC	CCTTCTCCTTGTCACTGGGCAC
Gdf5	NM_008109	ACAGCAGCGTGAAGTTGGAGGC	ACGGAGCGCACATCCAGCAA
Scx	NM_198885	CAGAGACGGCGGCGAGAACA	TGCTCTTGGGGACCTGCGCT
Ephb1	NM_001168296	ACTGTGCTTGTTCGTGACAGATGC	CTCTTCCCAGCGGGCCAAGC
Ntn4	NM_021320.3	AAGCGTGTTCCTGCCACCCG	CAGCTCCCCGCACAATCGCA
Mmp-10	NM_019471.3	GACCCCAGACAAATGTGATCCT	TTCAGGCTCGGGATTCCA

Supplementary Table S2. Genes up-regulated in FVB/N *Cd151*$^{-/-}$ compared to *Cd151*$^{+/+}$ glomeruli ($p < 0.05$)

Gene	Accession	Average Intensity *Cd151*$^{+/+}$	*Cd151*$^{-/-}$	Fold Change	p-value
Spon2	NM_133903.2	913.15	5008.025	5.48434	0.00211
Wasf1	NM_031877.2	93.5	171.575	1.835027	0.004264
4933407P14Rik	NM_199019.1	36.05	56.675	1.572122	0.004561
Lgals1	NM_008495.1	1270.575	2178.3	1.714421	0.006876
1110032E23Rik	NM_133187.2	62.025	136.275	2.197098	0.007983
Chn2	NM_023543.1	43.225	111.375	2.576634	0.008032
Timp1	NM_011593	96.325	165.275	1.715806	0.009132
Usp47	NM_133758.2	1366.4	1610.05	1.178315	0.01219
Mrpl33	NM_025796.2	2782.7	3268.65	1.174633	0.012727
Il18	NM_008360.1	16.575	45.2	2.726998	0.013862
Eno3	NM_007933.2	219.05	381.05	1.739557	0.014378
Slc6a12	NM_133661.3	33.675	108.95	3.235338	0.015083
Lrp5	NM_008513.1	96.225	127.3	1.322941	0.01621
Pcyox1	NM_025823	2807.1	3463.625	1.23388	0.019243
Rtel1	NM_001001882.2	84	97.8	1.164286	0.020011
Pdlim4	NM_019417.2	178.5	294.675	1.65084	0.020369
LOC237877	XM_111221.4	49.225	90.875	1.846115	0.020578
Metap1	NM_175224.4	1090.45	1432.9	1.314045	0.020794
Zbtb3	NM_133759.1	54.425	71.425	1.312356	0.020981

Prelp	NM_054077.3	82.225	128.65	1.564609	0.021247
Cp	NM_007752.2	180	248.625	1.38125	0.022102
LOC100047155	XM_001477537.1	108.825	178.95	1.644383	0.022115
Agrp	NM_007427.2	504.725	835.25	1.654862	0.02314
Cul4b	NM_028288.3	270.825	387.675	1.431459	0.024339
Nubp1	NM_011955.1	1431.525	2126.775	1.485671	0.025412
Nme1	NM_008704.2	7143.7	9796.05	1.371285	0.026093
C78339	NM_001033192.2	76.35	94.925	1.243287	0.026562
Ddah1	NM_026993	2378.325	3562.525	1.497913	0.027209
Slc22a8	NM_031194.3	8.875	73.125	8.239437	0.028713
Rsl1d1	NM_025546.2	659.7	724.8	1.098681	0.028771
Slc30a7	NM_023214.5	170.025	202.95	1.193648	0.029331
Noc3l	NM_021315.1	47.875	84.125	1.75718	0.02959
Apitd1	NM_027263.1	105.95	182.975	1.726994	0.029609
Htt	NM_010414.1	35.025	60.875	1.738044	0.030806
Srpk1	NM_016795.3	705.525	853.725	1.210056	0.030942
Ascc2	NM_029291.1	88.9	117.9	1.326209	0.031259
Pfdn1	NM_026027.3	16.075	41.525	2.583204	0.031373
Cib2	NM_019686.3	113.875	190.125	1.669594	0.031872
Hist1h4k	NM_178211.1	36.125	52	1.439446	0.03195
Capn7	NM_009796.2	277.375	385.875	1.391167	0.033429
Ankrd1	NM_013468.3	241.7	626.475	2.591953	0.033701
Ubie	NM_199477.2	141.525	305.425	2.158099	0.033834
Ccdc68	NM_201362.1	128.075	280.175	2.187585	0.035119
Ctps	NM_016748.1	1062.05	1515.775	1.427216	0.035587
4732471D19Rik	NM_176987.3	45.775	67.25	1.469143	0.035932
Sdsl	NM_133902.2	57.2	104.475	1.826486	0.036282
Ctsz	NM_022325.4	137.2	256.625	1.870445	0.037064
Lsm4	NM_015816.1	3959.925	5030.725	1.270409	0.037461
Olfml2b	NM_177068.3	261.475	524.475	2.005832	0.037622
Abcb1b	NM_011075.1	19.275	48	2.490272	0.038294
Ganc	NM_172672.2	525.025	698	1.329461	0.039291
C130032J12Rik	NM_178684.3	117.125	175.425	1.497759	0.040214
Aldh1a2	NM_009022.2	70.775	157.275	2.222183	0.040325
6720463M24Rik	NM_175265.4	22.975	46.925	2.042437	0.040885
Cops2	NM_009939.2	2907.975	3511.725	1.207619	0.040919
P2ry5	NM_175116.2	1117.525	1394.475	1.247824	0.041836
Ergic1	NM_026170.3	675.65	875.975	1.296492	0.042733
Lamc2	NM_008485.3	37.5	94.25	2.513333	0.043944
Zdhhc12	NM_001037762.1	875.975	1199.6	1.369445	0.044125
Nudt5	NM_016918.3	835.025	1025.425	1.228017	0.044617
Actl6a	NM_019673.1	9.625	39.675	4.122078	0.045301
Klhdc8b	NM_030075.1	18.6	49.175	2.643817	0.045418
Ints7	NM_178632.4	155.325	228.7	1.472397	0.045782
D5Ertd579e	NM_001081232.1	144.35	161.2	1.11673	0.046328
Lin54	NM_172714.1	864.125	1060.725	1.227513	0.046608
Hist1h2bm	NM_178200.1	1256.825	1521	1.210192	0.047425
Plek2	NM_013738.1	8.4	39.2	4.666667	0.04768
2810417H13Rik	NM_026515.2	3.8	49.725	13.08553	0.048474
Gabrb1	NM_008069.4	68.4	115.725	1.691886	0.048536
P2rx1	NM_008771.2	1103.325	1716.4	1.555661	0.048564
Fbxw5	NM_013908.4	3368.2	4388.225	1.30284	0.049242
Prnp	NM_011170.1	6246.175	10652.15	1.705388	0.04925

Supplementary Table S3. Genes down-regulated in FVB/N *Cd151*$^{-/-}$ compared to *Cd151*$^{+/+}$ glomeruli (p < 0.05)

Gene	Accession	Average Intensity *Cd151*$^{+/+}$	*Cd151*$^{-/-}$	Fold Change	p-value
Cd151	NM_009842.1	9080.525	-0.825	-999	0
Gdf5	NM_008109.1	73.025	13.7	-5.33029	0.00040035
Ccrk	NM_053180.2	76.525	57.375	-1.33377	0.00047559
1810014F10Rik	NM_026928.2	691.4	35.7	-19.3669	0.00104473
Stoml1	NM_026942.2	55.575	30.225	-1.83871	0.00105412
Arfl4	NM_025404.1	133.925	84.175	-1.59103	0.00128048
Tspan32	NM_020286.2	54.975	-2.775	-999	0.00145196
Dus3l	NM_144858.1	199.4	143.05	-1.39392	0.00221562
Tnfrsf22	NM_023680.2	251.45	74.825	-3.36051	0.00259574
Ephb1	NM_173447.2	1448.15	765.55	-1.89165	0.00294034
Dhps	NM_001039514.1	526.7	446.75	-1.17896	0.00316907
Frag1	NM_145583.2	80.75	60.875	-1.32649	0.00330005
Cdr2	NM_007672.1	3350.5	2337.65	-1.43328	0.00332108
4930438O05Rik	NM_030184.1	84.65	64.65	-1.30936	0.00348455
LOC100045005	XR_031422.1	3379.5	2242.95	-1.50672	0.00381625
Jund1	NM_010592.3	423.475	199.025	-2.12775	0.00387787
Lrrc45	NM_153545.1	188.55	146.325	-1.28857	0.00416849
BC066028	NM_001001180.2	42.225	9.6	-4.39844	0.00445089
Scx	NM_198885.2	227	87.975	-2.58028	0.00451969
2310002B06Rik	NM_181649.4	413.75	287.175	-1.44076	0.00462719
Lrrc1	NM_172528.2	634.875	415.725	-1.52715	0.00493553
Pitpnm1	NM_008851.1	86.775	69.7	-1.24498	0.00530035
Zfp414	NM_026712.2	151.65	99.5	-1.52412	0.0057679
Stx8	NM_018768.2	629.775	542	-1.16195	0.00591713
Mapk1ip1	NM_001045483.1	273.95	197.125	-1.38973	0.00677061
Il1rl1l	NM_010744.1	1093.375	877.5	-1.24601	0.00701077
Pfkl	NM_008826.2	1624.25	1175.7	-1.38152	0.00846755
Zbtb39	NM_198035.1	118.15	79.775	-1.48104	0.0092561
Med24	NM_011869.2	2285.675	1371.475	-1.66658	0.00984002
St6galnac6	NM_016973.2	67.325	33.625	-2.00223	0.01003419
Htatip	NM_178637.1	303.15	227.175	-1.33443	0.01043926
Nrip2	NM_021717.1	217.8	81.95	-2.65772	0.01059713
Bcl2l1	NM_009743.4	241.4	136	-1.775	0.0107301
Fkbp8	NM_010223.1	3095.95	2367.5	-1.30769	0.0107449
Ihpk2	NM_029634.1	165.625	54.1	-3.06146	0.01096123
Gsto2	NM_026619.1	75.225	35.725	-2.10567	0.01276501
Mett11d1	NM_001029990.1	658.975	530.8	-1.24148	0.01326912
Irf9	NM_008394.2	437.825	317.425	-1.3793	0.0134194
Rhbdf2	NM_172572.2	64.325	36.45	-1.76475	0.01369907
Trim46	NM_001039466.1	81.4	54.4	-1.49632	0.01437688
Sp6	NM_031183.1	43.225	24.725	-1.74823	0.01468138
Sf3b4	NM_153053.3	66.175	44.725	-1.4796	0.01470855
Jarid1b	NM_152895.1	55	42.625	-1.29032	0.01497226
Ffar2	NM_146187.3	72.35	18.3	-3.95355	0.01509752
Pps	NM_008916.2	1380	858.675	-1.60713	0.01521538
Loxl1	NM_010729.2	1141.55	742.8	-1.53682	0.01559306
Pltp	NM_011125.2	132.775	73.45	-1.80769	0.01562748
Pddc1	NM_172116.2	348.675	236.3	-1.47556	0.01595991

Zmat4	NM_177086.2	344.325	188.125	-1.8303	0.01645457
Evi1	NM_007963.1	109	69.1	-1.57742	0.01655893
Ctsa	NM_001038492.1	358.5	286.675	-1.25055	0.0167101
Scrn1	NM_027268.2	50.525	29.5	-1.71271	0.01678273
Nrd1	NM_146150.2	5662.5	4416.525	-1.28212	0.01686644
Mcc	NM_001033406.1	4196.9	2639.95	-1.58976	0.01711166
Vps25	NM_026776.3	3665.575	3199.025	-1.14584	0.01711543
Pias4	NM_021501.3	380.925	262.975	-1.44852	0.01728895
Ilvbl	NM_173751.3	1004.5	889.675	-1.12906	0.01743972
D7Bwg0611e	NM_027898.2	36.725	4.7	-7.81383	0.01750363
Rad23a	NM_009010.4	215.425	151.225	-1.42453	0.01775027
Ssbp4	NM_133772.1	454.625	288.6	-1.57528	0.01912805
Gpc1	NM_016696.3	7263.525	4748.7	-1.52958	0.01942314
Xylt2	NM_145828.2	296.55	243.55	-1.21761	0.01955261
Prnpip1	NM_080469.2	424.7	349.825	-1.21404	0.01961978
Plscr4	NM_178711.2	935.05	613.7	-1.52363	0.01967461
Retnlg	NM_181596.3	82.95	28.125	-2.94933	0.01973291
Inha	NM_010564.4	69.625	32.775	-2.12433	0.02009908
Sepw1	NM_009156.2	1707.2	1028.85	-1.65933	0.02045132
Rpl22	NM_009079.2	2531.35	1817.825	-1.39252	0.02046733
LOC100044324	XM_001471971.1	900.675	628.85	-1.43226	0.02054596
Otub1	NM_134150.2	624.95	492.175	-1.26977	0.0211242
Cd3d	NM_013487.1	45.575	15.475	-2.94507	0.02167872
Adora2b	NM_007413.4	307.225	223.35	-1.37553	0.02209584
Optn	NM_181848.3	1002.25	684.575	-1.46405	0.02217562
Wbscr22	NM_025375.1	670.05	581.2	-1.15287	0.02217948
Cbln4	NM_175631.3	96.75	41.775	-2.31598	0.02247628
Ubl7	NM_027086.1	2022.775	1656.575	-1.22106	0.02249082
Anxa4	NM_013471.1	1336.55	892.5	-1.49754	0.02252965
Mnt	NM_010813.2	57.725	23.5	-2.45638	0.0226808
Siat7f	NM_016973.1	229.9	118.925	-1.93315	0.02272428
Ankzf1	NM_026187.4	165.525	113.85	-1.45389	0.02276731
Senp1	NM_144851.3	79.4	47.925	-1.65676	0.02298466
Dok1	NM_010070.3	79.275	47.85	-1.65674	0.02329651
Pbx2	NM_017463.1	517.675	354.45	-1.4605	0.02342973
Ppp1r8	NM_146154.1	551.875	423.35	-1.30359	0.02432841
Eef2	NM_007907.1	1591.625	1012.7	-1.57166	0.02467927
Prkcb1	NM_008855.2	42.775	11.7	-3.65598	0.02503104
Sdc1	NM_011519.2	63.85	34.625	-1.84404	0.02529279
Rabif	NM_145510.1	423.5	301.425	-1.40499	0.02543967
Gmppa	NM_133708.3	285.5	248.375	-1.14947	0.02570374
Rnf44	NM_134064.1	4935.025	4085.85	-1.20783	0.02574612
Slc25a37	NM_026331.3	631.5	364.85	-1.73085	0.02576557
Trappc5	NM_025701.2	62.4	34.625	-1.80217	0.02578269
Prdm9	XM_911812.3	60.75	43.975	-1.38147	0.02587935
Pfkm	NM_021514.3	1262.35	906.225	-1.39298	0.02633984
Jun	NM_010591.1	3548.925	1675.775	-2.11778	0.02635025
Hoxb5	NM_008268.1	1064.375	653.1	-1.62973	0.02637197
Pgf	NM_008827.2	94.1	38.325	-2.45532	0.02676621
Zfp185	NM_009549.2	430.075	260.375	-1.65175	0.02718347
Dll4	NM_019454	208.2	72.55	-2.86975	0.02723799
Tnfrsf1a	NM_011609.3	352.175	250.575	-1.40547	0.02766843
2010001J22Rik	NM_001013022.1	90.45	52.625	-1.71876	0.02808741
Foxp1	NM_053202.1	263.025	195.175	-1.34764	0.02841333
2410081M15Rik	NM_028603.1	616.6	464.975	-1.32609	0.02891822

Snta1	NM_009228.1	565.075	445.7	-1.26784	0.02903195
AK129302	NM_001003916.1	277.5	200.2	-1.38611	0.02917632
2400001E08Rik	NM_025605.2	1337.95	1072.85	-1.2471	0.02927231
Smarcc2	NM_198160.1	417.1	320.15	-1.30283	0.0293805
Mtvr2	NM_023166	1077.7	790.975	-1.3625	0.02988094
Ltbp3	NM_008520.2	331.525	264.925	-1.25139	0.02998917
Mapkapk3	NM_178907.1	150.875	69.075	-2.18422	0.03074694
Magix	NM_018832.2	141.9	103.15	-1.37567	0.03086096
Brp17	NM_019999.1	2722.575	1808.575	-1.50537	0.03127211
Dyrk1b	NM_001037957.1	742.275	547.575	-1.35557	0.03134857
Zfp579	NM_026741.2	1117.225	813.025	-1.37416	0.03167165
Tug1	NR_002322.1	115.525	80.55	-1.4342	0.03171618
6430573F11Rik	NM_176952.4	105.575	47.125	-2.24032	0.03194674
Clec1a	NM_175526.1	103.85	59.875	-1.73445	0.03196523
Nr1h2	NM_009473.2	1421.2	1097.5	-1.29494	0.03196706
Rtp4	NM_023386.3	42	17.175	-2.44541	0.03225465
Ntng2	NM_133501.1	57.425	27.175	-2.11316	0.03234432
Mospd3	NM_030037.1	292.175	178.775	-1.63432	0.0324618
1110006G06Rik	NM_028661.1	260.775	178.225	-1.46318	0.03357466
4933439C20Rik	NM_001004146.1	1117.375	823.325	-1.35715	0.03418396
Ghdc	NM_031871.1	111.875	71.95	-1.5549	0.03433225
Ubap1	NM_023305.3	809.875	678.15	-1.19424	0.03435652
Gspt2	NM_008179.2	205.3	157.3	-1.30515	0.03440298
Yipf6	NM_207633.2	95.8	66.3	-1.44495	0.03453683
Gm114	NM_001033298.1	697.95	601.625	-1.16011	0.0345901
Taf5l	NM_133966.2	247.95	181.825	-1.36367	0.03531719
Snai1	NM_011427.2	126.175	71.325	-1.76902	0.03554617
Snca	NM_009221.2	327.95	134.7	-2.43467	0.03556926
Txnip	NM_001009935.2	9813.925	5961.95	-1.64609	0.03566088
Lmbr1l	NM_029098.3	280.9	199.425	-1.40855	0.03650645
A030010B05Rik	NM_030100	40.925	24.575	-1.66531	0.03663787
B930041F14Rik	NM_178699.3	903.425	668.95	-1.35051	0.03717492
LOC100045697	XR_031796.1	5807.725	5191.25	-1.11875	0.03726762
Npepl1	NM_213733.1	3022.275	2526.675	-1.19615	0.03764513
Junb	NM_008416.1	7596.425	3020.575	-2.51489	0.0377159
8430408G22Rik	NM_145980.1	1362.175	725.95	-1.8764	0.03778495
Tjap1	NM_028751.2	655.05	473.125	-1.38452	0.03833979
Ngb	NM_022414.2	55.225	22.575	-2.44629	0.03925148
Hba-a1	NM_008218.2	54769.4	26931.625	-2.03365	0.03984392
Hes1	NM_008235.2	1901.45	1179.125	-1.61259	0.03995203
Vamp4	NM_016796.2	1550.95	1024.5	-1.51386	0.04008572
Dusp15	NM_145744.2	255.15	95.8	-2.66336	0.04012412
Gdpd3	NM_024228.2	6226.925	4035.475	-1.54305	0.04060152
Ntn4	NM_021320.2	376.6	202.025	-1.86413	0.04067544
Zfp36	NM_011756.4	15898.92	8437.825	-1.88424	0.04067757
Ccdc28b	NM_025455.2	577.4	496.225	-1.16359	0.04075209
Acvr2b	NM_007397.2	1391.625	1056.725	-1.31692	0.04185702
Mobkl2c	NM_175308.4	50.05	42.125	-1.18813	0.04193526
Rnf5	NM_019403.3	2724.25	2107.9	-1.2924	0.04195608
Shisa3	NM_001033415.2	737.9	268.8	-2.74516	0.0421328
Ints6	NM_008715.2	38.475	16.3	-2.36043	0.04216053
Nfic	NM_026756.2	313.225	190.925	-1.64057	0.04218439
Sept1	NM_017461.2	43.175	8	-5.39688	0.0425559
Krba1	NM_133922.2	135.8	101	-1.34455	0.04286066
Cmtm8	NM_027294.1	1755.025	1073.85	-1.63433	0.04294303

Efcab4a	NM_001025103.1	1217.8	644.5	-1.88953	0.04344743
Clk2	NM_007712.2	643.15	498.275	-1.29075	0.04359061
B230317C12Rik	NM_019833.2	110.9	74.375	-1.49109	0.0446126
Hoxb2	NM_134032.1	399.625	335.275	-1.19193	0.04463566
Fcho1	NM_028715.2	89.375	43.5	-2.0546	0.04476396
Pde1a	NM_001009979.1	134.025	100.45	-1.33425	0.04493217
Mex3c	NM_001039214.3	45	17.375	-2.58993	0.04550276
Ckb	NM_021273.3	8025.575	5586.125	-1.4367	0.04553874
Cops7b	NM_172974.2	126.6	99.125	-1.27718	0.04584001
Suhw2	NM_177475.2	98.225	70.425	-1.39475	0.04584634
Itpr3	NM_080553.2	821.575	431.35	-1.90466	0.04610916
Spry4	NM_011898.2	344.55	211.675	-1.62773	0.04641986
Ankrd13d	NM_026720.1	56.05	28.275	-1.98232	0.04645498
Rgs4	NM_009062.3	751.1	426.3	-1.7619	0.04752539
Mea1	NM_010787.1	259.625	215.3	-1.20588	0.04779483
Smo	NM_176996.3	883.825	549.275	-1.60908	0.04796765
BC004728	NM_174992.2	826.475	719.05	-1.1494	0.04824104
Klf13	NM_021366.2	1659.575	939.025	-1.76734	0.04856825
LOC212390	XM_131360.3	396.375	247.375	-1.60232	0.04869439
Tmem132e	NM_023438.2	188.2	72.7	-2.58872	0.0487494
Phpt1	NM_029293.2	1622.95	1367.475	-1.18682	0.04884122
Pacsin2	NM_011862.2	360.3	321.55	-1.12051	0.04888485
Pbx1	NM_008783.1	223.7	161.05	-1.38901	0.04896532
Pcgf2	NM_009545.1	38.15	14.325	-2.66318	0.04945724
Fbxo9	NM_023605.2	907.35	762.2	-1.19044	0.04969794

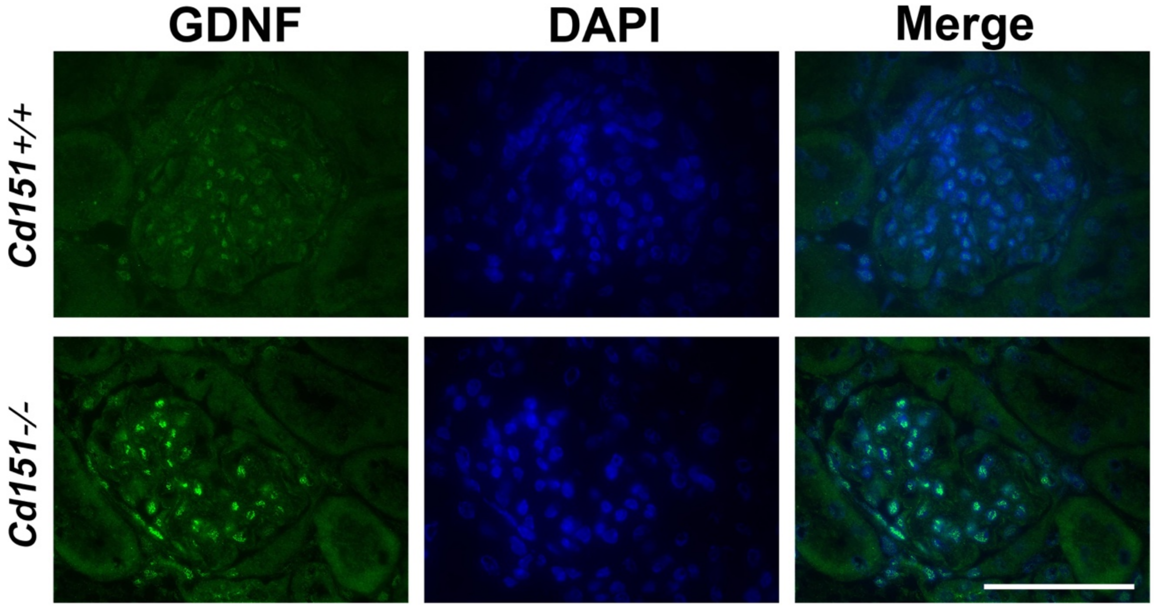

Supplementary Figure S1. Increased GDNF in the glomeruli of FVB/N *Cd151*$^{-/-}$ compared to *Cd151*$^{+/+}$ mice.

Immunofluorescence labelling for GDNF (green) demonstrated an increased labelling in the diseased glomeruli of 3-week-old FVB/N *Cd151*$^{-/-}$ mice compared to age-matched FVB/N *Cd151*$^{+/+}$ mice. This labelling correlated with DAPI (blue) indicating nuclear localisation. Representative images shown n=5 per mouse group. Original magnification x400. Bar: 50μM.

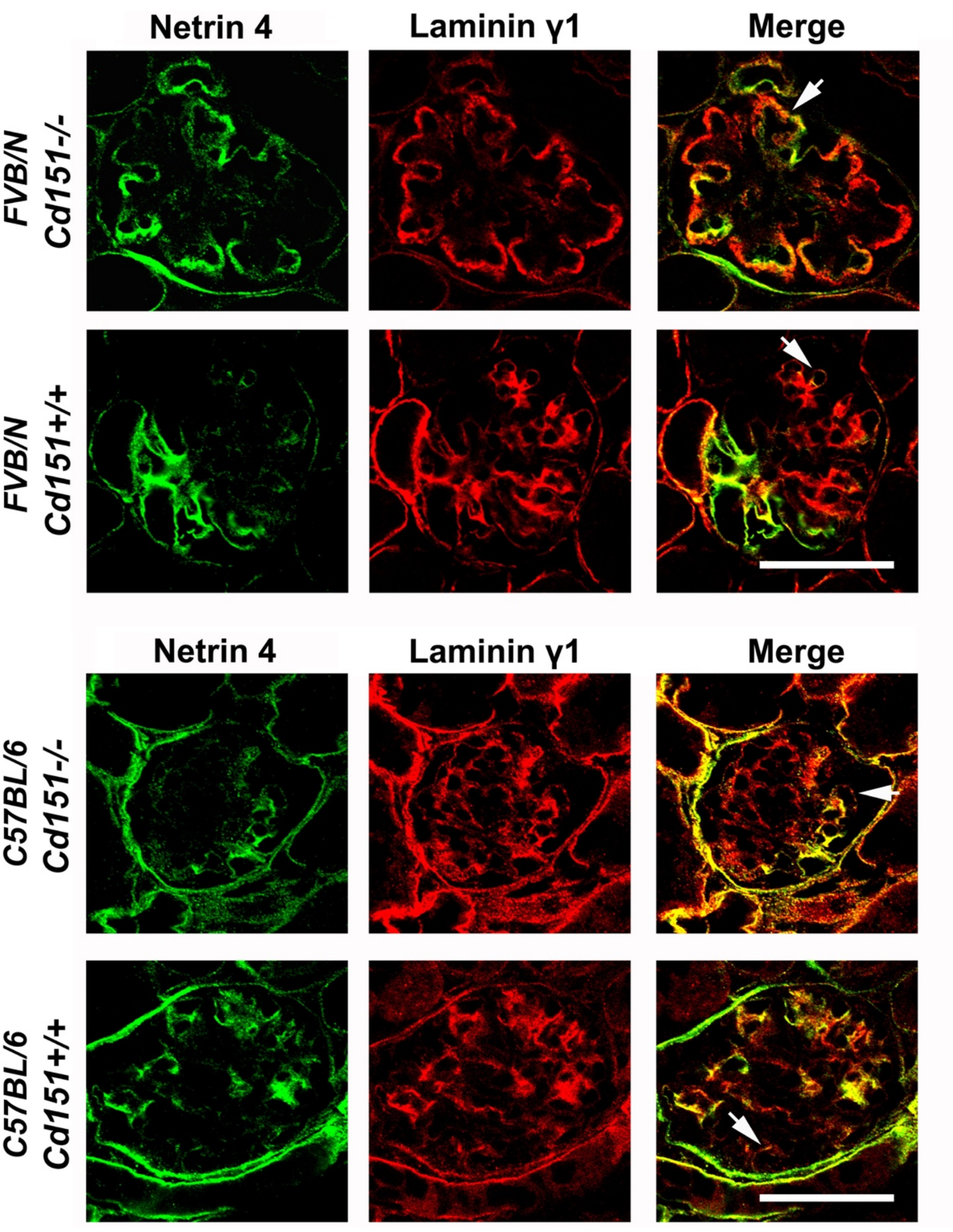

Supplementary Figure S2. Localisation of netrin 4 in the glomeruli of 3-week-old *Cd151*$^{-/-}$ and *Cd151*$^{+/+}$ mice.

Dual immunofluorescence labelling and confocal analysis of netrin 4 (green) and laminin γ1 (red). Netrin 4 fluorescence intensity was increased in the thickened GBM of FVB/N *Cd151*$^{-/-}$ mice (white arrows) as compared to FVB/N *Cd151*$^{+/+}$, C57BL/6 *Cd151*$^{+/+}$ and C57BL/6 *Cd151*$^{-/-}$ mice (white arrows). Representative images shown n=5 per mouse group. Original magnification x800. Bar: 50μm.

Uncropped gel images used to make Fig.3

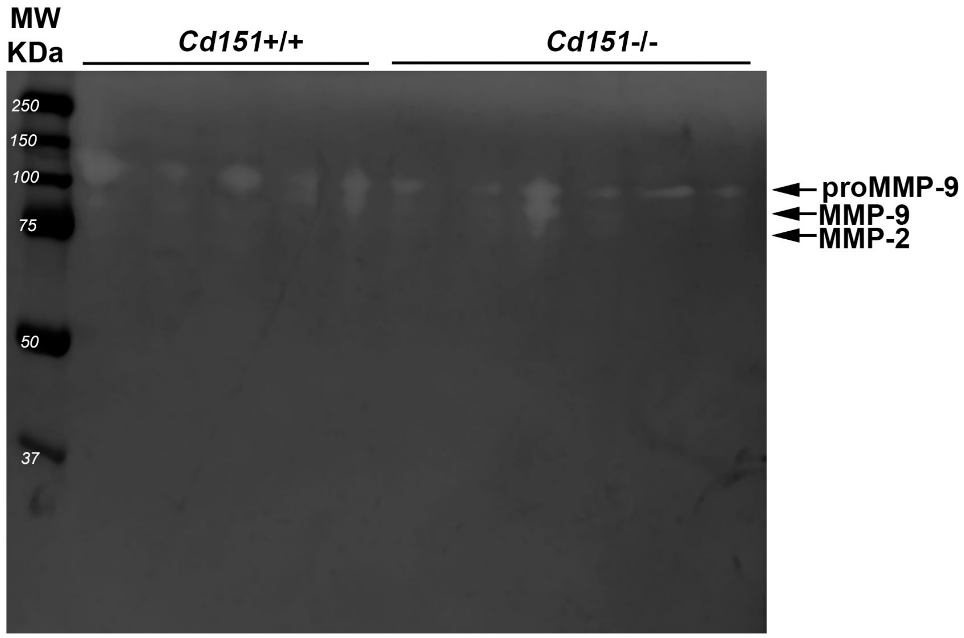

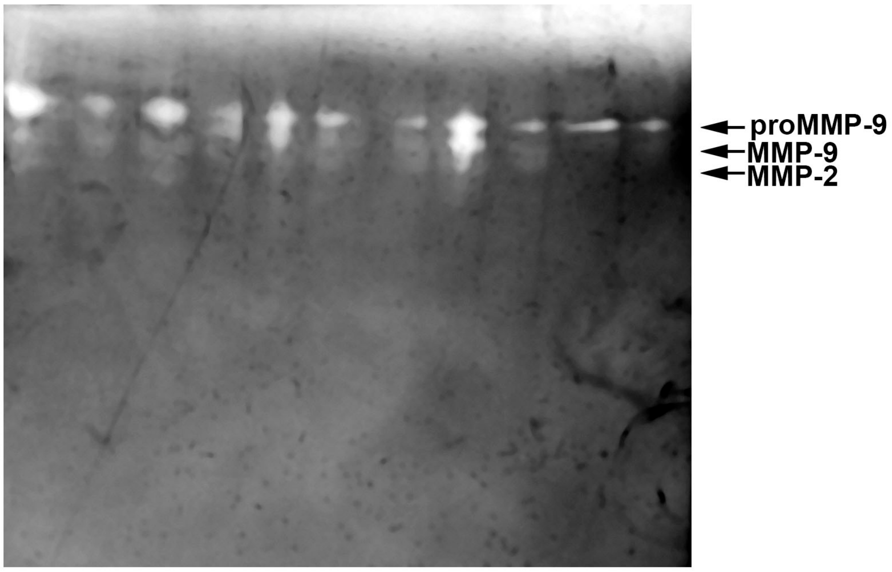

modified image after cutting out the lane
with MW marker from picture above and
adjustment with autocontrast
in Adobe Photoshop Elements editor

Uncropped mindin blot used in Figure 4C

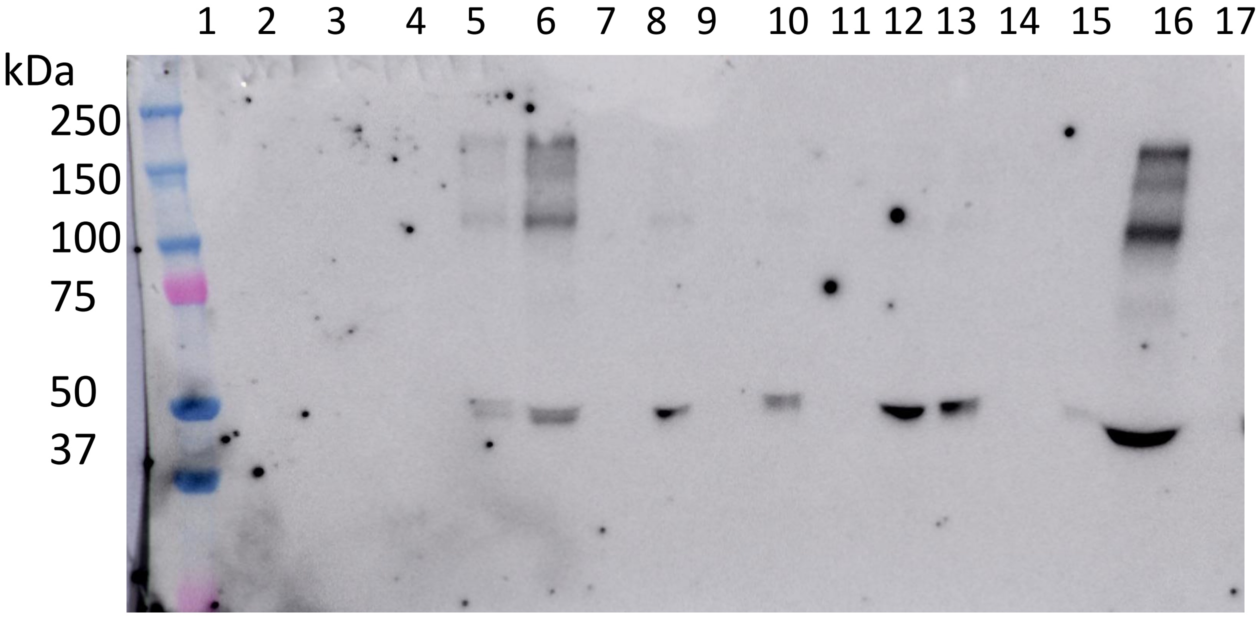

Mindin western Blot

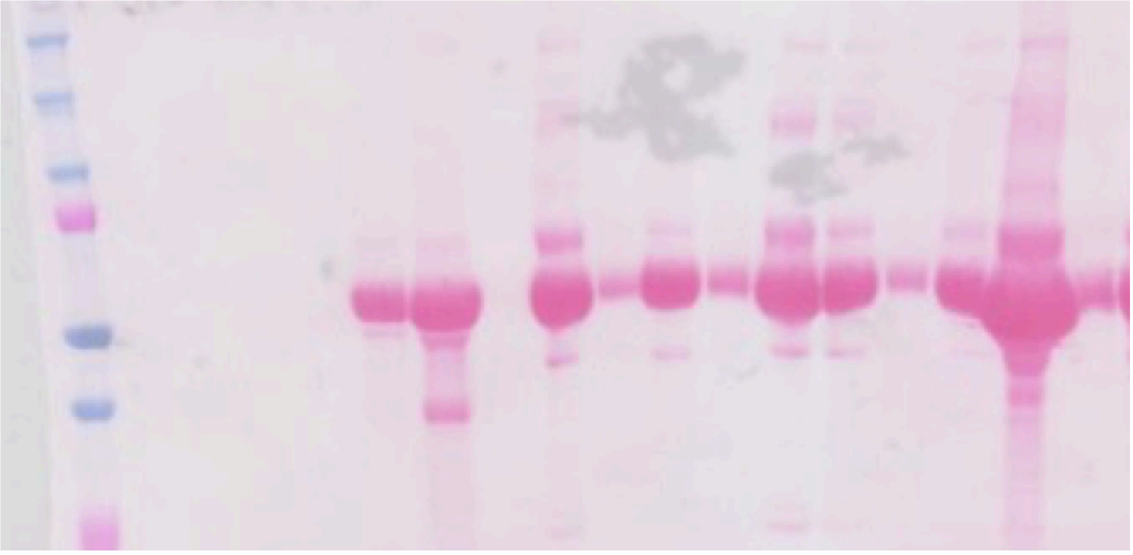

Ponceau
